# Resonant Rossby wave mechanism for extreme weather performs poorly in simple model test

**DOI:** 10.1126/sciadv.adp3054

**Published:** 2026-04-22

**Authors:** Todd A. Mooring, Marianna Linz

**Affiliations:** ^1^ Department of Earth and Planetary Sciences, Harvard University, Cambridge, MA 02138, USA.; ^2^ School of Engineering and Applied Sciences, Harvard University, Cambridge, MA 02138, USA.; ^3^ Reflective, San Francisco, CA 94105, USA.

## Abstract

Quasiresonant amplification (QRA) of quasistationary Rossby waves is a mechanism for mid-latitude extreme weather that has been repeatedly proposed but subjected to only limited testing. Here, we test QRA theory by attempting to create quasiresonant Rossby waves in an idealized general circulation model, in which we identify mean flow states expected to be suitable (and unsuitable) for the existence of quasiresonant Rossby waves and quantify the associated wave amplitudes. For mean flow conditions thought to be suitable for QRA, waves of the relevant wave number are instead found to be weaker than under conditions ostensibly unsuitable for QRA. This situation cannot robustly be changed by altering the definition of a QRA-suitable mean flow. These findings cast doubt on the value of QRA theory in its current form as an interpretive tool and more generally warrant caution in the use of purely two-dimensional theories and/or zonally averaged flows to explain tropospheric extreme event dynamics.

## Teaser

A proposed fluid dynamical explanation for certain extreme weather events appears not to work as advertised.

## INTRODUCTION

Heatwaves are devastating to human health (*1*, *2*), ecosystems (*3*, *4*), and crops (*5*) and have a disproportionate impact on lower income human populations (*6*, *7*). With global warming, extreme heat events are becoming more common (*8*) and long-lasting heatwaves are occurring with increasing frequency (*9*). Models misrepresent the temperature variability underpinning hot extremes (*10*, *11*) in some regions of the world, hindering projections of future heatwaves. Understanding the mechanisms that lead to increases in heatwaves is therefore a high priority for improving the understanding of the impacts of climate change.

Although much of the increase in extreme heat events can be attributed to mean warming (*12*, *13*), any change in the occurrence of weather patterns conducive to the development of heatwaves would modulate that increase. With a poleward shift of the jet stream, for example, an increase in extreme heat at higher latitudes would be expected (*14*, *15*). In mid-latitudes, heatwaves are often associated with large-amplitude quasistationary waves on the jet stream that buckle and remain in the same geographic location for an extended period of time [e.g., (*16*–*20*)]. These waves, known as Rossby waves, are fundamental to flows on a rotating sphere, but what determines the occurrence of such breaking events (or blocking) is quite complex (*21*, *22*). The most recent generation of comprehensive climate models shows improvement in representation of blocks, which form from such waves, but still notably underestimates North Atlantic winter blocking frequency and persistence (*23*). Models generally predict a decrease in the frequency of blocking (*24*) but also show a change in the relationship between heatwaves and blocking (*25*). A clear theoretical understanding of blocking and heatwaves should therefore improve attribution of present-day events and projections of future change.

A proposed dynamical mechanism for some heatwaves [and some other forms of extreme weather (*26*–*28*)] is quasiresonant amplification of Rossby waves, proposed in a specific quantitative form by Petoukhov *et al.* (*29*), which we hereinafter refer to as QRA. Stated in three sentences, the Petoukhov *et al.* (*29*) idea is that certain zonal-mean flow configurations constitute globe-circling waveguides that can meridionally trap quasistationary Rossby waves. The waveguide thus makes it easy for forcing to excite such waves, enabling the waves to grow to large amplitudes. Large-amplitude quasistationary waves constitute blocking events and favor the development of surface-level heatwaves.

**Fig. 1.**
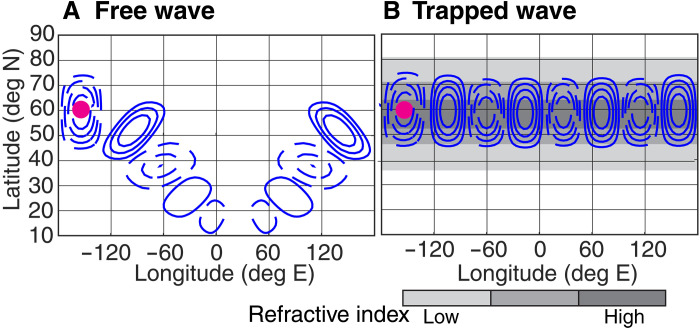
Free and trapped quasistationary Rossby waves. (**A**) Schematic of wave response to stationary forcing located at the pink dot with a weak background wind field. (**B**) Schematic of wave response to forcing located at the pink dot with a background wind field that creates a high refractive index for Rossby waves at the latitude colored in dark gray and a lower refractive index at the latitudes in lighter gray.

To illustrate the behavior of such a QRA waveguide, Fig. 1A shows a schematic of a freely propagating wave with weak background winds, while Fig. 1B shows a schematic of a trapped wave with a background flow that promotes QRA. The free wave propagates along a great circle into the tropics, where the weak background easterly winds cause it to dissipate [e.g., (*30*)]. The trapped wave cannot propagate outside the QRA waveguide, and so an initial perturbation follows the waveguide along a line of constant latitude. The wave activity is trapped and does not dissipate, so the magnitude is larger than in the free wave case. Both panels show teleconnection patterns, with a nonlocal response to a local perturbation, but only the second case is associated with persistent, amplified waves at a fixed latitude and their associated surface weather consequences.

QRA has been used to explain the existence of observed extreme events, especially summertime heatwaves (*31*–*35*). Furthermore, as the waveguide and trapped wave are present at all longitudes, multiple simultaneous extreme weather events in different regions may result (*36*). Surface temperature patterns associated with QRA increase in frequency in climate model projections of global warming (*37*), and other diagnostic work indicates that QRA has become more important for the development of Northern Hemisphere mid-latitude heatwaves in recent decades (*38*). Although the general idea of Rossby wave propagation being shaped by waveguides has been discussed for many years (*39*) and QRA builds on the linearized barotropic vorticity equation well known to geophysical fluid dynamicists, the full set of assumptions Petoukhov *et al.* (*29*) needed to quantitatively develop QRA theory is quite complex and the applicability of insights gained from linear analyses of the barotropic vorticity equation to large-amplitude waves in the real atmosphere has been questioned (*40*).

As the first step of efforts to better understand and test QRA theory, we embarked on a quest to produce quasiresonant Rossby waves in an idealized general circulation model (GCM). Unexpectedly, the quest failed. Specifically, we found that the existence of waveguides defined on the basis of 300-hPa zonal winds (as in previous work) was associated with reduced amplitude of the ostensibly quasiresonant waves, even as those waves were more contained meridionally as expected with waveguides. This finding is a demonstration by counterexample that the core qualitative prediction of QRA theory is not necessarily correct. In an attempt to better understand these results, we also explored alternative waveguide definitions based on zonal winds at other pressure levels or on Ertel potential vorticity (hereinafter EPV) gradients evaluated on isentropes. The existence of waveguides based on 200-hPa zonal winds is, in some cases, associated with elevated wave amplitudes—but far less robustly than the existence of waveguides based on 300-hPa winds, which are associated with reduced wave amplitudes. While the existence of “EPV waveguides” defined using our approach can also be associated with higher-amplitude waves, this result is not robust to small changes in the potential temperature at which mean state EPV gradients are evaluated.

Last, the relationships between near-surface temperature extremes and the existence of putative (300-hPa zonal wind) waveguides are complex and not especially consistent with QRA theory. We therefore submit that the underlying assumptions of QRA theory need to be more critically interrogated and that existing QRA-based diagnostic work needs to be reevaluated. Furthermore, our findings on both QRA and EPV waveguides suggest that caution is warranted in using purely two-dimensional fluid dynamical theories and/or zonally averaged mean states to explain certain aspects of Rossby wave dynamics in the actual three-dimensional troposphere.

## RESULTS

### Theoretical background

QRA theory as laid out by Petoukhov *et al.* (*29*) is an application of the barotropic vorticity equation on the sphere. The most important thing to understand about the theory is that it evaluates the potential for Rossby waves to become meridionally trapped—i.e., prevented from propagating poleward or equatorward—by using the Wentzel-Kramers-Brillouin approximation to analyze the propagation of linear waves on a zonal wind mean state that depends on latitude but not longitude or time. The dispersion relation for free Rossby waves under these conditions is

$$\omega =\frac{\mathrm{UK}}{\text{cos}\phi }-\frac{\beta_{M}K}{K^{2}+l^{2}}$$ (1)

where ω is the wave angular frequency; U is the mean state zonal wind; ϕ is the latitude; K and l are dimensional zonal and meridional wave numbers, respectively; and $\beta_{M}$ is a function of U, ϕ, and the Earth rotation rate Ω (Materials and Methods). For the present study, we are interested only in quasistationary waves, and for ω=0, Eq. 1 implies that the (dimensional) meridional wave number l and (dimensionless) zonal wave number k are related according to

$$a^{2}l^{2}=k_{s}^{2}-k^{2}$$ (2)

where a is the Earth radius and

$$k_{s}^{2}=\frac{2\Omega a{\text{cos}}^{3}\phi }{U}-\frac{{\text{cos}}^{2}\phi }{U}\frac{\partial^{2}U}{\partial \phi^{2}}+\frac{\text{sin}\text{ϕcos}\phi }{U}\frac{\partial U}{\text{∂ϕ}}+1$$ (3)

which will be referred to as the squared stationary wave number.

For free waves to propagate meridionally, we need $l^{2}>0$, and for a given $k^{2}$, the latitudes at which propagation can occur are therefore determined by $k_{s}^{2}$. Let us suppose that we have a mean flow $k_{s}^{2}$ and choose k such that there are two latitudes at which $l^{2}=0$, a region of $l^{2}>0$ in between, and $l^{2}<0$ outside this region. Such an $l^{2}>0$ region should function as a waveguide from which quasistationary waves of zonal wave number k cannot easily escape (Fig. 1), subject to some quantitative caveats (Materials and Methods). The characteristics of the background flow thus lead to regions that effectively have low refractive indices flanking a region of high refractive index (Fig. 1B). The inability of waves to escape from such a waveguide suggests that it should be relatively easy for thermal or orographic forcing to excite waves therein and cause them to grow to a large amplitude. This hypothesized ease of generating forced waves is the physical basis for the suggestion of Petoukhov *et al.* (*29*) that waveguides promote the development of large waves and, by extension, surface extreme weather.

An alternative conceptualization of waveguides is that—because Rossby waves are waves on potential vorticity gradients—waveguides can be defined as jumps or sharp meridional gradients in mean state potential vorticity [e.g., (*39*–*41*)]. For analyses of Rossby waves in three-dimensional atmospheres, this idea can be quantified using gradients of EPV computed on isentropes (*42*, *43*). The latitude of a waveguide can then be taken as the latitude of the maximum magnitude of the gradient in (the natural logarithm of) the EPV. Because in this study we are interested in hemispherically coherent waves, we will define our EPV waveguides using zonal-mean EPV fields.

Perhaps the greatest conceptual difference between the QRA and EPV waveguide perspectives is that the existence of a QRA waveguide is a fundamentally binary matter: Such a waveguide does not naturally have an associated magnitude; one simply either exists or does not. In contrast, an EPV waveguide has a magnitude (the magnitude of the maximum gradient of the natural logarithm of the EPV), but there is no theoretically motivated threshold that separates EPV waveguide existence from nonexistence. In practice, we reduce (scalar) EPV waveguide magnitudes to (binary) existence statuses by tuning a threshold EPV waveguide magnitude separating larger- and smaller-magnitude EPV waveguides such that QRA and EPV waveguides are equally common in our GCM simulations. Further details are presented later in the paper.

### Model, experiment setup, and analysis approach

To test QRA theory, we need a model that is as simple as possible without being either a mere restatement of the theory or a further working-out of the consequences of its assumptions. An idealized dry primitive equation GCM (Materials and Methods and fig. S1) with carefully constructed thermal forcing fits this description: Because QRA theory is derived from the barotropic vorticity equation, complex physical processes such as a water cycle, realistic radiation, or sophisticated land surface physics should not be needed. By the same token (and despite its intended use for the diagnosis of reanalysis data and realistic GCM simulations), QRA theory ignores the three-dimensional nature of the atmosphere and thus must parameterize baroclinic eddy effects on the hypothesized quasiresonant waves. To investigate whether realistic eddies or other three-dimensional aspects of the atmosphere such as finite static stability (*44*) somehow break QRA theory, it is therefore desirable to conduct the test in a three-dimensional model. Last, to test QRA theory, we want to examine the relationship between wave properties and the mean state using mean states that do and do not have QRA waveguides but that are otherwise very similar. Comparing waves on very different mean states would make it harder to argue that the presence or absence of a waveguide is the relevant difference between them.

We performed two experiments with this model. In the first experiment, which we refer to as wave6_heat, we supplement the model’s basic Newtonian relaxation thermal forcing with additional specified heating aimed at exciting a stationary wave. This heating has zonal wave number 6 and a meridional structure and location chosen to efficiently excite quasistationary waves in QRA waveguides of the sort that the model tends to create (Materials and Methods and fig. S2). The second experiment (referred to as no_heat) is identical to the first experiment, except without the wave-6 specified heating—in no_heat, we are therefore examining whatever quasistationary waves appear without making any explicit attempt to excite them. This experiment can be considered a robustness check or a control.

Again, the main conceptual claim of QRA theory is that the existence of a QRA waveguide as defined above tends to promote the development of large-amplitude quasistationary waves. Here, we outline our main test of this claim—see Materials and Methods for more details:

1) For a given experiment and hemisphere, split the model output into a large number of nonoverlapping time windows.

2) For each window, determine whether the time-mean zonal-mean flow contains a QRA waveguide expected to trap zonal wavenumber 6 on the basis of the criteria enumerated in (*33*).

3) For each window, compute suitable time and meridional averages of the meridional wind field and then evaluate the amplitude of the zonal wave-6 quasistationary wave therein.

4) By combining the results of steps 2 and 3, calculate distributions of wave amplitude conditional on the existence (or nonexistence) of a QRA waveguide.

5) Compare the wave amplitude distributions from step 4 to see whether they are distinct.

6) Repeat steps 1 to 5 but with the wave amplitudes calculated using windows that are lagged in time (forward or backward) relative to the windows used to check for QRA waveguides. This accommodates the possibility that the wave amplitude takes time to respond to the formation of a waveguide—and the possibility that waveguide formation is promoted or inhibited by preexisting waves of a particular amplitude.

Note that the above-described analytical approach does not make any use of the QRA forcing formula proposed by Petoukhov *et al.* (*29*) as their equation S1c and restated in a simpler form as equation 5 in (*33*). This is because we question its physical validity: Specifically, the thermal part of the forcing formula is a function of the wave component of the upper tropospheric temperature field. To our knowledge, a clear theoretical justification for this approach has never been presented in the literature, and because the forcing so defined is itself a function of the (temperature) wave amplitude, we are concerned that any quasistationary wave theory that uses such a forcing as input may be circular.

However, we view the validity of the specific Petoukhov *et al.* (*29*) forcing formula as a less fundamental issue than whether QRA waveguides themselves actually act to promote the growth of large-amplitude waves by trapping wave activity. We submit that our experimental design is able to test whether or not they do—by focusing directly and exclusively on the covariation of wave-6 amplitude and mean state in our simulations, we eliminate the need to additionally assume the validity of the proposed forcing formula.

### QRA waveguides at 300 hPa are associated with suppressed wave amplitudes

**Fig. 2.**
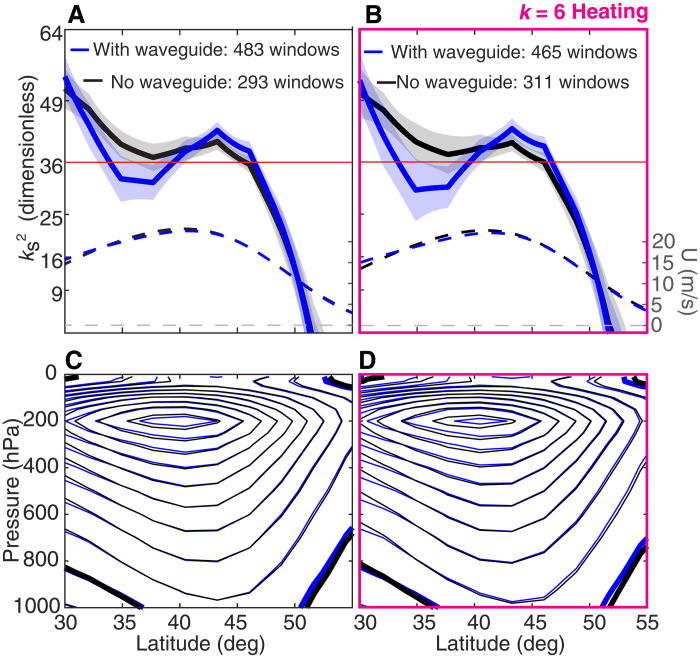
Squared stationary wave numbers and their underlying zonal jets. (**A** and **B**) Composite $k_{s}^{2}$ (solid lines, left vertical axis) and zonal-mean zonal wind (dashed lines, right vertical axis) profiles at 300 hPa for 15-day windows with a waveguide present (blue) and without a waveguide present (black). (**C** and **D**) Zonal-mean zonal winds composited over 15-day windows with (blue) and without (black) a waveguide present. The contour spacing is 3 m/s, with the zero contour marked in bold. Panels (A) and (C) show results for no_heat, while panels (B) and (D) show results for wave6_heat. Numbers of windows for which a waveguide is and is not present are listed at the top of (A) and (B). Error bars are 1 standard deviation.

First, we examine the squared stationary wave number ($k_{s}^{2}$) profiles of our experiments to illustrate the effect of internal variability on this property of the mean state and confirm that our objective waveguide identification algorithm is working properly (Fig. 2). The plotted $k_{s}^{2}$ profiles are computed from 300-hPa zonal winds, consistent with previous work, and are each composited over hundreds of 15-day windows following the procedure mentioned in the previous section. In qualitative agreement with our discussion of the requirements for QRA, the with-waveguide composite profiles have prominent local maxima near 43° latitude with $k_{s}^{2}>36$ but also regions of $k_{s}^{2}<36$ both poleward and equatorward of this latitude. If QRA theory is correct, this implies trapping of k=6 quasistationary Rossby waves in the meridional region bounded by the two $k_{s}^{2}=36$ latitudes. In contrast, the no-waveguide composite profiles cross $k_{s}^{2}=36$ only once in the latitude range of interest, consistent with the absence of a second such latitude as required for a QRA waveguide. Although there are quantitative differences between the waveguide composites for the wave6_heat and no_heat experiments, they are qualitatively quite similar. This suggests that the addition of wave-6 heating does not radically transform the mean flow, easing comparisons between the two experiments. Furthermore, the same basic phenomena are seen in both hemispheres of both experiments (fig. S5). This supports the idea that our model runs are indeed long enough for our results to be physically robust and not simply random manifestations of internal variability.

The zonal-mean zonal wind composite profiles for windows with and without QRA waveguides are shown in Fig. 2 [dashed lines in Fig. 2 (A and B) and solid lines in Fig. 2 (C and D)] and highlight an important fact about QRA theory—specifically, the zonal-mean zonal winds can be very similar in both cases. This is because the formula for $k_{s}^{2}$ (Eq. 3) depends on both the first and second meridional derivatives of the zonal-mean zonal wind, and so quite subtle differences in the wind profiles result in distinct $k_{s}^{2}$ profiles that differ as to whether they contain waveguides. We also examine the three-dimensional spatial structures of the quasistationary waves (supplementary text and figs. S3 and S4). The most important result of this analysis is that the structure of the waves in the run without additional specified heating is quite similar to the structure in the run where such heating is applied, demonstrating that our heating excites waves of the kind that are already favored by the zonal-mean flow field. The structures are also largely independent of waveguide status, although the surface pressure field structures (fig. S3, A to D) are suggestive of a slightly greater degree of meridional confinement when a QRA waveguide is present.

**Fig. 3.**
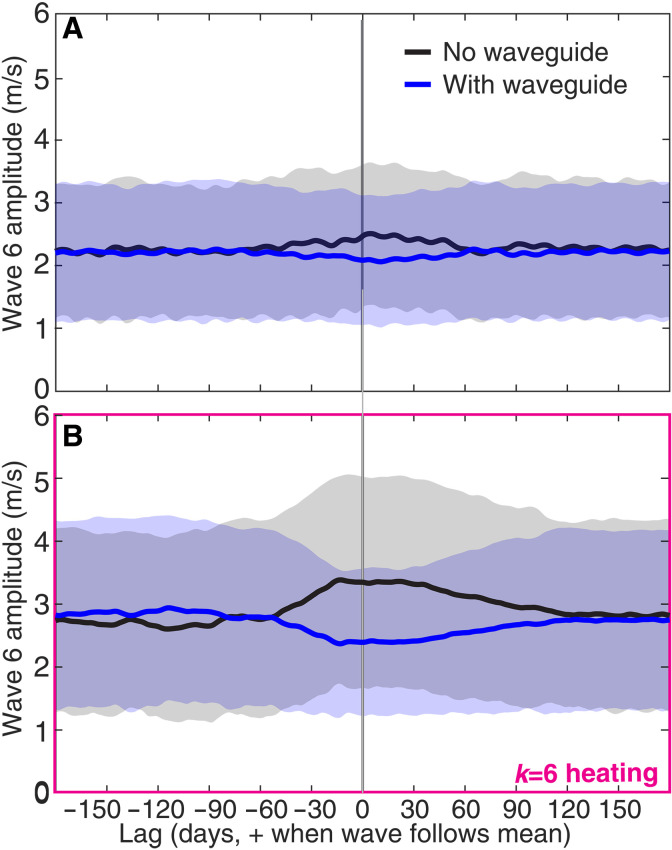
Reduced wave amplitudes in the presence of 300-hPa QRA waveguides. Amplitude of quasistationary zonal wave number 6 in the 300-hPa meridional wind field for 15-day windows with (blue) and without (black) a 300-hPa QRA waveguide in the no-heating experiment [no_heat, (**A**)] and the experiment with wave-6 specified heating [wave6_heat, (**B**)]. The amplitude is shown as a function of lag in days—the sign convention for lag is that positive (negative) lags indicate that the wave amplitude is evaluated using an averaging window after (before) the window for which waveguide status is determined. Error bars are 1 standard deviation across all relevant windows.

We now directly address our main research question: What is the relationship between QRA waveguide existence and wave amplitude? We made Fig. 3 following the procedure laid out in the previous section, reducing the with-waveguide and no-waveguide wave-6 amplitude distributions to their means and standard deviations. A clear pattern emerges: In both experiments, for lags near zero, the existence of a waveguide is associated with lower amplitude of the quasistationary wave 6. We consider the results from wave6_heat to pose a particular challenge to QRA theory—although one might be able to dismiss the no_heat results as a consequence of a lack of appropriate thermal forcing, such an argument cannot (in our view) explain why the existence of a waveguide is associated with weaker waves even when thermal forcing designed to excite quasiresonant waves is present.

Our core result of a weakened wave 6 in the presence of a QRA waveguide based on 300-hPa zonal winds is extremely robust at small to moderate values of the lag. For lags 0 to +30 days, the mean strength of wave 6 is weaker with a waveguide than without one. This is true in both hemispheres of both experiments (fig. S6) and when we raise the length of the averaging windows to 30 or 60 days. It is still true when we quantify the strength of the waves using a single latitude (43.25°) of 300-hPa meridional wind instead of the 37.5° to 57.5° meridional mean we usually use or use a cross-window median instead of a cross-window mean to summarize the wave amplitude distributions. All of these quantification approaches implicitly assume that a difference in the mean or median is an adequate paradigm for describing the dependence of the wave-6 amplitude distribution on waveguide status. At least in principle, more complex distributional shape changes are possible—we therefore also tried quantifying wave amplitudes using means ± 1 standard deviation (Fig. 3) and upper and lower quartiles (fig. S6). All of the aforementioned results still hold.

In additional rounds of robustness testing, we attempted to repeat parts of the above analysis but with the $k_{s}^{2}$ profiles defined on the basis of zonal winds at 200 or 500 hPa instead. This turns out to result in substantial reductions in the number of QRA waveguides found in the simulations—the number of waveguides at 200 (500) hPa is at most 8% (4%) as large as at 300 hPa depending on the exact experiment, hemisphere, and window length analyzed. With 60-day averaging windows, QRA waveguides become virtually absent at 200 and 500 hPa: Searching across all eight relevant combinations of experiment, hemisphere, and pressure, at most 3 of 194 windows have a waveguide. Because there are many fewer time windows with waveguides at 200 or 500 hPa than with waveguides at 300 hPa, the analytical approach outlined in the previous section and implemented in Fig. 3 and fig. S6 is not usable. Instead, as detailed in the supplementary text and tables S1 to S6, we develop and use a permutation technique to investigate whether the relationships between waveguide existence and wave amplitude are consistent with the idea that waveguides promote the growth of large-amplitude (300-hPa meridional wind zonal wavenumber 6) waves.

The permutation technique confirms the negative relationship between 300-hPa QRA waveguide existence and wave-6 amplitude (tables S1 and S4) and provides virtually no support for the idea that 500-hPa QRA waveguides are associated with large-amplitude wave-6 waves (tables S2 and S5). In contrast, the relationship between 200-hPa QRA waveguide existence and wave-6 amplitude is more complex and equivocal: There is some evidence that waveguide existence is associated with larger-amplitude waves, particularly for the wave6_heat experiment analyzed with 15-day windows. However, the successes of QRA theory with waveguides defined using 200-hPa zonal winds are far less robust than its failures with waveguides defined using 300-hPa zonal winds—the large sensitivity of the results to the exact level at which the zonal jet is evaluated itself bodes ill for the physical significance of QRA theory’s waveguide diagnostic.

### EPV waveguides lack a robust relationship with wave amplitudes

We also explore the possibility that the observed wave behaviors are better explained in terms of the EPV waveguide concept outlined above and elaborated in Materials and Methods. It is not a priori obvious on which isentrope the EPV should be evaluated for comparison to waves defined using the 300-hPa meridional wind field. We therefore begin our EPV waveguide analyses by following the recent study of White and Admasu (*43*), who compare 300-hPa meridional wind quasistationary waves to EPV-based waveguide diagnostics computed on the 345 (330) K isentrope for summer (winter).

**Fig. 4.**
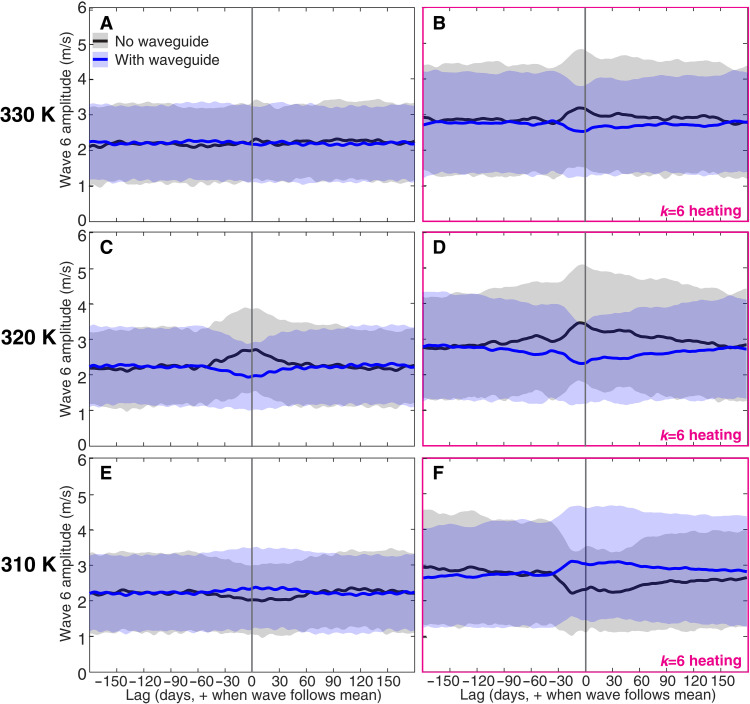
Relationships between wave amplitudes and EPV waveguides. As in Fig. 3 but with waveguide existence determined from zonal-mean EPV fields on three different isentropes. Results for 330, 320, and 310 K are shown in (**A)** and (**B**), (**C**) and (**D**), and (**E**) and (**F**), respectively, while no_heat (wave6_heat) results are shown in (A), (C), and (E) [(B), (D), and (F)].

Performing an analysis similar to the one underlying Fig. 3 for both 345 and 330 K (i.e., 15-day averaging windows, individual-window wave-6 amplitudes computed as spatial means over 37.5° to 57.5°, and wave-6 amplitude distributions summarized by means and means + 1 standard deviation), we find that for wave6_heat the presence of a (EPV) waveguide is again associated with lower amplitude of the quasistationary wave 6 at all lags 0 to +30 days (Fig. 4B). For no_heat the 345 K results are identical to those for wave6_heat, while at 330 K (Fig. 4A), the apparent suppression of wave amplitude by waveguide presence extends only out to a lag of +20 (+19) days if the amplitude distribution is summarized by its mean (mean + 1 standard deviation). (Results for the remaining lags to +30 days are hemisphere-dependent and therefore not robust to internal variability.)

These initial results do not support the idea that EPV waveguides promote the development of large-amplitude quasistationary wave-6 waves. However, our idealized GCM simulations have colder middle and upper tropospheres (fig. S7) than the actual Northern Hemisphere summer—upon which many QRA observational studies focus—does (*45*). Because one would expect wave dynamics to be more a function of atmospheric pressure than of the overall vertically and horizontally averaged atmospheric temperature, it may be appropriate to instead use EPV at lower values of potential temperature. We therefore repeat the above-described EPV waveguide analysis at 320, 315, and 310 K—the mean pressure at 320 (315) K and 30° to 70° is 274 (321) hPa in both idealized GCM simulations, similar to the 300 hPa claimed to be relevant to quasiresonant Rossby waves in the actual atmosphere.

In both experiments, the presence of an EPV waveguide at 320 K is yet again associated with a lower wave-6 amplitude for lags 0 to +30 days (Fig. 4, C and D). However, the sign of this relationship abruptly varies with potential temperature such that the presence of waveguides at 315 and 310 K is instead (finally) associated with higher wave-6 amplitudes for lags 0 to +30 days (Fig. 4, E and F). More generally, these results are highly robust to other relatively arbitrary changes in the data analysis procedure: The lag 0 to +30 days results for 320, 315, and 310 K of both experiments—and also 345 and 330 K of wave6_heat—are left unchanged when the averaging window is raised to 30 or 60 days, the wave amplitude is instead quantified at 43.25°, and/or the wave-6 amplitude distribution is characterized by its median and upper quartile. For no_heat there is more sensitivity to such analysis details at 330 K, but at 345 K, waveguide existence is still generally associated with weaker wave-6 waves. Finally, we performed additional sensitivity tests using the permutation technique developed to analyze 200- and 500-hPa QRA waveguides (supplementary text and tables S7 to S10). These tests are used to analyze several more isentropes and in general suggest that EPV waveguides at 330 to 355 K are associated with weaker wave-6 waves or have no clear relationship with their amplitude, while 320 to 325 K (310 to 315 K) waveguides are if anything associated with weaker (stronger) waves. The difference in 310 to 315 K and 320 to 325 K waveguide responses is particularly prominent in the wave6_heat experiment.

## DISCUSSION

We have shown that QRA theory’s most direct prediction—that the existence of a waveguide promotes the development of large-amplitude upper tropospheric waves—is apparently not generally correct. However, the core motivation for QRA theory is to explain surface-level extremes and perhaps the existence of a QRA waveguide is nevertheless associated with surface extremes for other as-yet-unknown reasons. Although our model cannot simulate extreme events that involve moisture or complex land surface processes, we expect (and QRA theory implicitly assumes) that purely dry dynamical processes play an important role. For completeness, we therefore also use our experiments to examine the relationship between waveguide existence and near-surface temperature extremes.

The results of this analysis (supplementary text and fig. S8) give almost no support to the idea that 300-hPa QRA waveguides increase the overall frequency of extreme temperatures in their vicinities, as one might expect from QRA theory. We find that the presence of 300-hPa QRA waveguides is not unambiguously associated with more extreme events but that waveguides are associated with suppressed hot extremes in the vicinity of the waveguides. Waveguides are also associated with more cold extremes in the waveguide region. This asymmetry between hot and cold extremes is consistent with the idea that jet properties are critically important for temperature skewness (*15*, *46*) but is distinct from the predictions made by QRA theory.

Returning to our main result, our finding that 300-hPa QRA waveguides appear to suppress wave activity is curious—particularly in the context of the reported success of very similar versions of QRA theory in many other studies. Broadly speaking, there are four main areas of difference between our work and the existing QRA literature:

1) Our idealized GCM has highly simplified atmospheric physics relative to realistic climate models or the actual Earth as represented in reanalysis.

2) The mean state and variability of the idealized GCM are presumably less “Earthlike” than those of realistic climate models and reanalyses.

3) Our analytical approach does not attempt to make use of the wave forcing formula proposed by Petoukhov *et al.* (*29*).

4) We experiment with using 200- and 500-hPa zonal winds as metrics of the zonal-mean flow, in addition to the 300-hPa zonal winds typically used.

Inasmuch as the requirements for a QRA waveguide are fully embodied in the formal definition provided by Kornhuber *et al.* (*33*) and our GCM’s mean flows satisfy that definition, the idealized physical parameterizations and mean states of our model should not break QRA theory, the idealized GCM should be a valid tool for testing the theory, and the apparent association of 300-hPa QRA waveguides with weaker waves must count as evidence against it.

Alternatively, based especially on the analysis of the wave6_heat experiment with waveguide status diagnosed from 15-day mean 200-hPa zonal winds, one might try to argue that QRA theory is sound but that for the idealized GCM’s mean states the zonal-mean zonal winds are most appropriately evaluated at 200 hPa. The less impressive results for no_heat and with longer window lengths could perhaps be dismissed as due to the absence of wave-6 thermal forcing and too few windows with 200-hPa waveguides to reliably quantify their effects, respectively. However, it remains undeniable that in our experiments QRA theory’s failures with zonal winds evaluated at 300 hPa are far more robust than its successes with zonal winds evaluated at 200 hPa.

Why might QRA theory work less well in our idealized GCM than in the real terrestrial atmosphere? A preliminary comparison of the middle and upper tropospheric thermal structures of the idealized GCM simulations to ERA5 reanalysis (*45*) reveals that the static stability of the idealized GCM is higher than that of the actual Northern Hemisphere summer extratropics (fig. S7) often analyzed in QRA studies. The effective static stability difference between the idealized GCM and realistic climate models or the real world may be even higher than implied by this comparison because of the absence of latent heating in the idealized GCM [e.g., (*47*, *48*)]. Perhaps our simulations are simply too statically stable for their wave behavior to be adequately explained by barotropic theory—but if that is the case, QRA theory still needs to be amended to more clearly define the static stability limits on its validity.

On the other hand, the static stability in the idealized GCM simulations is similar to that found in Northern Hemisphere winter and other studies argue that the barotropic vorticity equation can be a viable representation of winter dynamics [e.g., (*49*–*51*)]. It is therefore not at all obvious that the higher static stability of our simulations should break QRA theory. If QRA waveguide theory with the zonal-mean zonal winds evaluated at 300 hPa is indeed valid and applicable to our simulations, then to obtain the results we actually obtained the true effective forcing for wave-6 quasistationary waves must somehow be strongly anticorrelated with the existence of 300-hPa QRA waveguides. It is difficult to see how this could be, particularly for our simulation with specified time-independent wave-6 heating. The initial work on QRA theory (*29*) argued that the development of apparent QRA events was driven primarily by variations in the mean flow rather than by the proposed QRA forcing term reaching large values—to the extent that this argument is valid and carries over to our idealized GCM, it supports our choice to neglect possible time variations in forcing. Furthermore, to the extent that quasistationary wave development is instead forced by baroclinic transient waves, this is still indicative of a problem with an aspect of the broader QRA theory [which assumes that such transient waves damp the quasistationary waves (*29*)].

We therefore propose three possible explanations—which are not mutually exclusive—for QRA’s apparent success in the real world despite its poor performance in our idealized test:

1) Some form of QRA theory is valid, but only under additional conditions satisfied in the real atmosphere but not in our idealized model.

2) Some form of QRA theory is valid, but mean state differences between the real atmosphere and the idealized model require adjustments to the waveguide diagnosis approach (e.g., evaluation of the zonal winds at a higher altitude) to obtain positive results with the idealized model.

3) Statistically real but fundamentally noncausal relationships exist between large waves and QRA waveguides, which ultimately arise from other more general constraints on the relationships between waves and mean flows. For example, mean states that promote the development of large waves in the real atmosphere might coincidentally tend to satisfy the QRA 300-hPa waveguide criteria, while mean states that promote the development of large waves in the idealized model might tend not to. It is also possible that large waves essentially geometrically favor the existence of QRA waveguides in the real world (*52*) but not in the idealized model.

Unfortunately, all three possibilities bode ill for the practical utility of QRA theory in its current form. If the last possibility is correct, then QRA theory’s successes are at least partly an illusion and its explanatory power is diminished accordingly. If the first (and/or second) possibility is correct, unless and until the additional validity conditions can be identified (and/or the details of the waveguide diagnosis approach can be better justified), it will inevitably be unclear whether they remain satisfied (and/or appropriate) as the climate changes. This makes existing attempts to use QRA theory for future projections [e.g., (*37*, *53*)] and to analyze extended past time periods [e.g., (*54*, *55*)] difficult to interpret.

Although our main focus in this study is to test QRA theory in a form very similar to what was proposed by Petoukhov *et al.* (*29*), we also experimented with an alternative waveguide definition based on EPV gradients. While this alternative could have been expected to lead to better results (*40*, *43*), it did not: Instead, the relationship between wave amplitudes and EPV-based waveguides was sometimes highly sensitive to the exact isentrope on which the EPV gradient was evaluated and (barring a compelling physical argument for the use of particular isentropes) was therefore arguably basically arbitrary and physically meaningless.

It is not immediately obvious how to reconcile the notable sensitivity of our findings to waveguide evaluation height with other published results that support the value of a barotropic or single-isentrope interpretation of quasistationary wave dynamics. Perhaps these two-dimensional perspectives are simply inadequate for understanding our specific problem (namely, the wave-6 amplitude response to variations in the zonal jet). Alternatively, our choice to compute the mean flow as an Eulerian zonal mean may not be physically reasonable (*42*, *52*). We must also emphasize that our work should not be seen as undermining the value of the larger waveguide concept or the importance of amplified waves for extreme events. Meridional gradients of zonal wind do of course affect the propagation of Rossby waves, and waveguides and their relationships with extreme events are explored in detail in a recent review (*39*). The proposed physical mechanisms and core qualitative predictions of QRA theory and our EPV-based modification thereof are a mere subset of the broader waveguides/extremes ideas, and other possible waveguides/extremes theories are not addressed here. We consider the exploration of alternative definitions of the mean flow from which waveguides are computed to be a promising direction for future work (*42*, *43*, *52*).

## MATERIALS AND METHODS

### Rossby wave dispersion relation

The unforced barotropic vorticity equation in spherical coordinates may be written as

$$\frac{D\left(\zeta +f\right)}{\mathrm{Dt}}=\left[\frac{\partial }{\partial t}+\frac{u}{a\text{cos}\phi }\frac{\partial }{\partial \lambda }+\frac{v}{a}\frac{\partial }{\partial \phi }\right]\left[\zeta +2\text{Ωsin}\theta \right]=0$$ (4)

where ζ is the relative vorticity, f is the Coriolis parameter, and λ and t are the longitude and time, respectively. Because the flow is nondivergent, we can represent the velocity field using a stream function ψ

$$\left(u,v\right)=\left(-\frac{1}{a}\frac{\text{∂ψ}}{\text{∂ϕ}},\frac{1}{a\text{cos}\phi }\frac{\text{∂ψ}}{\partial \lambda }\right)$$ (5)

The definition of relative vorticity then implies

$$\zeta =\frac{1}{a^{2}\text{cos}\phi }\left[\frac{1}{\text{cos}\phi }\frac{\partial^{2}\psi }{\partial \lambda^{2}}+\frac{\partial }{\partial \phi }\left(\text{cos}\phi \frac{\text{∂ψ}}{\text{∂ϕ}}\right)\right]=\nabla^{2}\psi$$ (6)

By combining Eqs. 4 and 6, we can form a nonlinear equation for the evolution of ψ. Because we want to analyze the dynamics of waves, we linearize about a zonally symmetric mean flow U=U(ϕ) to get an equation for the stream function anomaly $\psi^{\prime }$

$$\begin{array}{c} \left[\frac{\partial }{\partial t}+\frac{U}{a\text{cos}\phi }\frac{\partial }{\partial \lambda }\right]\nabla^{2}\psi^{\prime }+\\ \left[\frac{2\Omega }{a^{2}}-\frac{1}{a^{3}\text{cos}\phi }\frac{\partial }{\partial \phi }\left(\frac{1}{\text{cos}\phi }\frac{\partial \left(U\text{cos}\phi \right)}{\partial \phi }\right)\right]\frac{\partial \psi^{\prime }}{\partial \lambda }=0 \end{array}$$ (7)

Following (*29*), we opt to seek solutions to Eq. 7 by first putting it on a Mercator projection, which is defined as

$$\left(x,y\right)=\left[a\lambda ,a\text{ln}\left(\frac{1+\text{sin}\phi }{\text{cos}\phi }\right)\right]$$ (8)

By also defining

$$\tilde{U}=\frac{U}{\text{cos}\phi }$$ (9)

and

$$\beta_{M}=\frac{2\Omega {\text{cos}}^{2}\phi }{a}-\frac{\partial }{\partial y}\left(\frac{1}{{\text{cos}}^{2}\phi }\frac{\partial \left(\tilde{U}{\text{cos}}^{2}\phi \right)}{\partial y}\right)$$ (10)

and multiplying Eq. 7 by ${\text{cos}}^{2}\phi$, we are able to rewrite it as

$$\left[\frac{\partial }{\partial t}+\tilde{U}\frac{\partial }{\partial x}\right]\left[\frac{\partial^{2}}{\partial x^{2}}+\frac{\partial^{2}}{\partial y^{2}}\right]\psi^{\prime }+\beta_{M}\frac{\partial \psi^{\prime }}{\partial x}=0$$ (11)

Note that for arbitrary U, Eq. 11 cannot be expected to have simple analytic solutions. We therefore assume that

$$\psi^{\prime }\left(x,y,t\right)=\psi_{a}\left(x,y,t\right)\text{exp}\left[i\theta \left(x,y,t\right)\right]$$ (12)

Defining

$$\left(K,l,\omega \right)=\left(\frac{\partial \theta }{\partial x},\frac{\partial \theta }{\partial y},-\frac{\partial \theta }{\partial t}\right)$$ (13)

and assuming that θ varies sufficiently rapidly in space and time relative to $\psi_{a}$, K, l, and ω, we can substitute Eqs. 12 and 13 into Eq. 11 and get

$$\left[-i\omega +\tilde{U}\mathrm{iK}\right]\left[-K^{2}-l^{2}\right]\psi_{a}\text{exp}\left(i\theta \right)+\beta_{M}\mathrm{iK}\psi_{a}\text{exp}\left(i\theta \right)=0$$ (14)

which can be readily manipulated to yield Eq. 1.

### Waveguide detection algorithms

The qualitative explanations in the main text of how waveguides are supposed to work and the features they must have omit many quantitative and implementation details. Because the underlying QRA theory assumes that the mean state flow is time-independent, we perform our waveguide diagnosis on 15-, 30-, and 60-day–mean zonal-mean zonal wind fields. Only the 15-day means are presented in the main text. We use these zonal wind fields to evaluate $k_{s}^{2}$ according to the definition in Eq. 3. Because of the multiple meridional derivatives involved in evaluating $k_{s}^{2}$, it is advantageous to have a smooth and quasicontinuous zonal wind field for use as input to the $k_{s}^{2}$ calculation. We achieve this by using Matlab’s spline functionality to interpolate the zonal-mean zonal wind fields from the model-native ~2.8° grid to a 0.01° grid. We evaluate the relevant meridional derivatives using finite differences.

We then search the $k_{s}^{2}$ profiles for QRA waveguides. Although the physical bases of waveguide criteria such as a bound on the minimum allowed width are described in (*29*), for consistency with more recent diagnostic work, we instead simply implement our waveguide detection code using the list of waveguide requirements given by Kornhuber *et al.* (*33*). More specifically, we require the following:

1) The existence of two turning point latitudes (i.e., latitudes at which $l^{2}=0$).

2) $l^{2}>0$ between these latitudes.

3) Both of the turning point latitudes located in the 30° to 70° latitude range.

4) U>0 between the turning point latitudes and within the region extending to 2.5° north (south) of the northward (southward) turning latitude.

5) $l_{\text{max}}^{2}$, the largest value of $l^{2}$ achieved in the $l^{2}>0$ region, must satisfy $10^{-13}\leq l_{\text{max}}^{2}\leq 10^{-12}$ m^−2^.

6) The waveguide width (i.e., the distance between the two turning point latitudes) must be ≥2°.

All but the fourth of these requirements are taken without modification from (*33*)—the Kornhuber *et al.* (*33*) paper asserts that U>0 is required between and “in the vicinity of” the turning points but never quantitatively defines “vicinity.” We therefore chose 2.5° as our definition of “vicinity” after inquiring with K. Kornhuber. Our experiments never produce more than one waveguide candidate satisfying the above requirements for each $k_{s}^{2}$ profile searched, so the waveguide spacing constraint used in (*33*) is irrelevant to our work.

A similar approach is used for the EPV waveguides. The EPV (here denoted q) is defined as

$$q=-g\frac{\partial \theta }{\partial p}\left(\zeta +f\right)$$ (15)

where p is the pressure, θ is now the potential temperature, and g is the strength of gravity. The EPV is computed from idealized GCM output using instantaneous 6-hourly horizontal wind and ∂θ/∂p fields defined on isentropes and then averaged in longitude and over time periods of 15, 30, and 60 days. The magnitude of the natural logarithm of the EPV gradient ∣∇logq∣ is given by

$$|\nabla \text{log}q|=\left|\frac{1}{q}\frac{\partial q}{a\partial \phi }\right|$$ (16)

and, for a given isentrope and hemisphere of interest, we search the 15° to 70° latitude range for the maximum value of ∣∇logq∣ (denoted ${|\nabla \text{log}q|}_{\text{max}}$) and its associated latitude $\phi \left({|\nabla \text{log}q|}_{\text{max}}\right)$. Analogous to our treatment of the zonal wind fields for the QRA waveguide analysis, we spline-interpolate zonal-mean EPV fields onto a 0.01° grid to enable the accurate calculation of the meridional derivative.

We posit that $\phi \left({|\nabla \text{log}q|}_{\text{max}}\right)$ and ${|\nabla \text{log}q|}_{\text{max}}$ are metrics of EPV waveguide location and strength, respectively. Because these quantities are defined for each and every 15-, 30-, or 60-day window of interest, they cannot be used directly to separate windows “with” and “without” EPV waveguides in a manner analogous to what is done for QRA waveguides. Reducing a collection of ${|\nabla \text{log}q|}_{\text{max}}$ values to binary existence statuses therefore requires that we posit a threshold value of ${|\nabla \text{log}q|}_{\text{max}}$ that separates waveguide (larger ${|\nabla \text{log}q|}_{\text{max}}$) and no-waveguide (smaller ${|\nabla \text{log}q|}_{\text{max}}$) cases. Rather than adopt a specific threshold value from previous work [e.g., (*42*, *43*)], we instead tune the threshold such that the number of EPV waveguides is the same as the number of 300-hPa QRA waveguides—this is a reasonable approach because we are ultimately interested in the relationship between wave amplitude and waveguide status and not in estimating an absolute waveguide frequency. Because the number of (300 hPa) QRA waveguides varies as a function of experiment, hemisphere, and window length, the selected ${|\nabla \text{log}q|}_{\text{max}}$ thresholds vary with experiment, hemisphere, window length, and isentrope on which ∣∇logq∣ is evaluated.

Note lastly that our EPV waveguide search region (15° to 70°) is extended equatorward relative to the 30° to 70° constraint on QRA waveguides. We made this choice to reduce the frequency with which $\phi \left({|\nabla \text{log}q|}_{\text{max}}\right)$ is simply the equatorward edge of the search region. For the 315 K isentrope in particular, it is still relatively common (tens of percent of averaging windows) to have $\phi \left({|\nabla \text{log}q|}_{\text{max}}\right)=15{}^{\circ}$. However, the $\phi \left({|\nabla \text{log}q|}_{\text{max}}\right)=15{}^{\circ}$ phenomenon is notably less common at 310 K, and our ultimate results for both isentropes are essentially identical—we therefore conclude that this computational pathology is not physically problematic in practice.

### General circulation model

Our idealized GCM is a derivative of the well-known Geophysical Fluid Dynamics Laboratory spectral model (*56*). The model experiments performed for this paper used T42 horizontal resolution, for which the associated Gaussian grid has a spacing of ~2.8°. The vertical structure of the model consists of 40 evenly spaced sigma levels and there is no topography. The surface boundary layer parameterization is Rayleigh drag exactly as in (*56*). Other basic model parameters such as the planetary radius and gas constant are appropriate for Earth.

The main thermal forcing of the model is Newtonian relaxation

$${\left.\frac{\partial T}{\partial t}\right|}_{\text{newt}}=-k_{T}\left(T-T_{\text{eq}}\right)$$ (17)

where ${\left.\frac{\partial T}{\partial t}\right|}_{\text{newt}}$ is the temperature tendency resulting from Newtonian relaxation, the thermal damping rate $k_{T}$ is a function of latitude and (sigma) model level according to the formula in (*56*), T is the (location- and time-dependent) temperature, and $T_{\text{eq}}$ is the latitude- and pressure-dependent Newtonian relaxation temperature.

The $T_{\text{eq}}$ field is the most distinctive and important feature of our model. We generated it using an iterative algorithm very similar to the one created by Chang (*48*). The iteration process consists of a succession of 600-day model runs, which we index by j. The $T_{\text{eq}}$ field is updated over the series of model runs according to the formula

$$T_{\text{eq}}^{j}=T_{\text{eq}}^{j-1}-\frac{2}{3}\left({\bar{T}}^{j-1}-T_{\text{clim}}\right)$$ (18)

where $T_{\text{eq}}^{j}$ and $T_{\text{eq}}^{j-1}$ are the $T_{\text{eq}}$ fields used to force runs j and j−1, ${\bar{T}}^{j-1}$ is the (interhemispherically symmetrized time-mean zonal-mean) temperature over the last 400 days of run j−1, and $T_{\text{clim}}$ is a latitude- and pressure-dependent target temperature distribution. In physical terms, this algorithm raises (lowers) $T_{\text{eq}}$ when ${\bar{T}}^{j-1}$is too cold (hot) relative to $T_{\text{clim}}$—the prefactor $\frac{2}{3}$ is meant to enhance the convergence of the iteration process by reducing the size of the $T_{\text{eq}}$ change in a single iteration and therefore reducing the probability of overcorrecting a $\bar{T}$ error. We initialized the iteration process by choosing $T_{\text{eq}}^{1}=T_{\text{clim}}$ and ultimately concluded that $T_{\text{eq}}^{18}$ yielded $k_{s}^{2}$ profiles of sufficient quality to force the production wave6_heat and no_heat experiments.

We created the $T_{\text{clim}}$ field for our experiments by concocting a $k_{s}^{2}$ structure that included a k=6 waveguide centered at 40° latitude with a width of 10°. We then used Eq. 3 to derive an associated 300-hPa zonal wind field and (after assuming a vertical structure for this zonal jet) used gradient wind balance to derive a zonally symmetric temperature distribution consistent with this desired waveguiding jet. (We do not consider the exact details of this scheme especially important, as the $k_{s}^{2}$ profiles shown in Fig. 2 are hardly exact copies of the waveguide assumed in constructing $T_{\text{clim}}$ and therefore our success in creating a flow with useful 300-hPa $k_{s}^{2}$ profiles is partly a coincidence.) Specified wave-6 thermal forcing was not used during the iteration process. Note also that because we chose $T_{\text{clim}}$ to be interhemispherically symmetric and because we interhemispherically average the ${\bar{T}}^{j}$ during the iteration process, all $T_{\text{eq}}^{j}$ are interhemispherically symmetric, and we can therefore expect the climates of our production experiments to also be interhemispherically symmetric—this feature is important for our uncertainty analyses. In fig. S1, we show zonal-mean zonal wind fields for both experiments—the use of wave-6 specified heating apparently has little effect on the mean state.

### Wave-6 thermal forcing

QRA theory as laid out by Petoukhov *et al.* (*29*) assumes that there is thermal or orographic forcing to excite the quasiresonant Rossby waves. It is questionable whether our model’s Newtonian relaxation alone can be an adequate forcing. To see this, let us express the temperature T in Eq. 17 as the sum of a time-mean zonal-mean $\bar{T}$ and a time- and longitude-dependent anomaly $T^{\prime }$: $T=\bar{T}+T^{\prime }$. Equation 17 can then be written as

$${\left.\frac{\partial T}{\partial t}\right|}_{\text{newt}}=-k_{T}\left(\bar{T}-T_{\text{eq}}\right)-k_{T}T^{\prime }$$ (19)

The $k_{T}\left(\bar{T}-T_{\text{eq}}\right)$ term is zonally symmetric and therefore not able to excite zonal waves, while the $k_{T}T^{\prime }$ term simply damps $T^{\prime }$—which includes all zonal wave components of the temperature field—back to zero at a rate $k_{T}$.

To guarantee the presence of forcing to excite waves, we therefore also use specified wave-6 heating in the wave6_heat experiment. This time-independent forcing can be written as

$$\begin{array}{c} {\left.\frac{\partial T}{\partial t}\right|}_{\text{newt}}\\ =\left\{\frac{1\;K}{86,400\;s}\right.{\left(\frac{p}{1000\;\text{hPa}}\right)}^{\kappa }\text{exp}\left(\frac{p-1000\;\text{hPa}}{250\;\text{hPa}}\right)\\ 0\\ \text{cos}\left(3.6\left(|\phi |-43.25^{\circ }\right)\right)\text{cos}\left(6\lambda \right)\;18.25^{\circ }<|\phi |<68.25^{\circ },p\geq 50\;\text{hPa}\\ \text{elsewhere} \end{array}$$ (20)

where pressure p has units of hPa, latitude ϕ and longitude λ now have units of degrees, and $\kappa =\frac{R}{c_{p}}=\frac{2}{7}$ is the ratio of the gas constant to the specific heat capacity of air at constant pressure.

As illustrated in fig. S2, the strength of this heating peaks near the surface at ~1 K/day. The decrease in heating with height is qualitatively consistent with a diagnostic study based on (Northern Hemisphere winter) European Centre for Medium-Range Weather Forecasts analyses (*57*), but we deliberately chose to make our specified heating relatively weak in comparison to this observation-based estimate. By choosing a weak forcing, we can keep the zonal-mean states of no_heat and wave6_heat relatively similar—this is helpful because, as detailed below, the horizontal structure of ${\left.\frac{\partial T}{\partial t}\right|}_{\text{newt}}$ has been chosen to efficiently excite waves on the no_heat mean state. We do not consider the use of a weak forcing in wave6_heat to be a major impediment to testing QRA theory—given that the theory is basically linear [compare (*58*)], quasiresonant waves probably do not need to have any particular amplitude, and we should be able to reliably quantify the mean state dependence of even weak waves by running our experiments sufficiently long.

We conclude this discussion of the wave-6 thermal forcing by justifying its horizontal structure. Given that we wanted (and succeeded in creating) 300-hPa wave-6 waveguides in no_heat, we need to force at k=6 to possibly get a quasiresonant wave in wave6_heat—hence, the cos(6λ) term in Eq. 20. Inspection of a preliminary plot of 300-hPa $k_{s}^{2}$ based on a subset of no_heat output suggested that (for a k=6 free Rossby wave), $l^{2}$ would take on its maximum value in the waveguide—i.e., reach $l_{\text{max}}^{2}$—near 43.25° latitude. Furthermore, we found $a^{2}{l^{2}}_{\text{max}}\approx 5.2$. This implies a meridional wavelength scale on the Mercator projection of $L_{\text{max}}\approx 17,600$ km. By linearizing the Mercator projection (Eq. 8) about 43.25° and then using it to “deproject” back to the sphere, we can ultimately show that a wave with $a^{2}l^{2}\approx 5.2$ at 43.25° has a meridional length scale of ~115° on the sphere. Our choice of $\text{cos}\left(3.6\left(|\phi |-43.25^{\circ }\right)\right)$ has a comparable meridional length scale (100°) and places the peak forcing strength near the $l^{2}$ maximum in the waveguide. The forcing therefore has a similar horizontal structure to the free Rossby waves that the waveguide is intended to trap—as desired for efficient excitation of forced quasiresonant waves.

### Waveguides and wave amplitudes: Detailed methods

Here we more rigorously state the primary analysis procedures used for investigating the dependence of wave amplitude on the existence or nonexistence of waveguides. We describe the QRA analysis procedure first:

1) For a given experiment and hemisphere, we divide the (instantaneous, 6-hourly) 300-hPa zonal wind data into nonoverlapping N-day windows where N is 15, 30, or 60 days. We can index the windows by i=0,1,…,M−1, where M is the total number of windows and window i starts at day X+iN.

2) For each of the M N-day windows, we compute the time-mean zonal-mean zonal wind at 300 hPa. Then we determine whether this zonal wind profile entails a $k_{s}^{2}$ profile that constitutes a waveguide for quasistationary wave 6 according to the arguments sketched in the main text and detailed in Materials and Methods.

3) For the same experiment and hemisphere, we quantify the size of the quasistationary waves in terms of the amplitude of the wave-6 component of a time-averaged area-weighted meridional mean meridional wind field at 300 hPa. The meridional mean is taken over 37.5° to 57.5° latitude in the hemisphere of interest, and for consistency with the zonal wind/waveguide analysis, we apply an N-day time average to the area-weighted meridional mean meridional wind data before computing the wave-6 amplitude. Furthermore, instead of just computing wave-6 amplitudes for the group of M windows that begin at days X,X+N,X+2N,…,X+(M−1)N (i.e., the same windows used for the waveguide diagnosis in step 2) we also perform the analysis for lagged windows beginning at days X+D,X+N+D,X+2N+D,…,X+(M−1)N+D. Lagged wave amplitude calculations are of interest because they allow us to investigate whether waveguide existence is informative not merely about current (D=0) wave amplitude but also wave amplitude in the future (D>0) or in the past (D<0).

4) We combine the results from steps 2 and 3 to form distributions for each lag D of wave-6 amplitude conditional on the existence (or nonexistence) of a waveguide.

5) We summarize the conditional distributions in terms of their means and standard deviations and thereby compare the waveguide and no-waveguide conditional distributions. These comparisons are our main result. As mentioned in Results, we also conducted limited sensitivity tests quantifying the jet structures at 200 and 500 hPa. These sensitivity tests led us to conclude that the QRA waveguide/wave amplitude analysis as described here could not be repeated at these additional pressure levels, motivating the additional permutation test–based analysis approach.

The main EPV analysis procedure is quite similar:

1) For a given experiment and hemisphere, we divide the (instantaneous 6-hourly) EPV data on the 345 K isentrope into nonoverlapping N-day windows.

2) For each of the M N-day windows, we compute the time-mean zonal-mean EPV at 345 K and then derive its associated $\phi \left({|\nabla \text{log}q|}_{\text{max}}\right)$ and ${|\nabla \text{log}q|}_{\text{max}}$ values.

3) We select a threshold value of ${|\nabla \text{log}q|}_{\text{max}}$ such that the total number of 345 K EPV waveguides for the experiment and hemisphere of interest is the same as the number of (300-hPa) QRA waveguides for that experiment and hemisphere.

4) For the same experiment and hemisphere, we quantify the amplitude of wave-6 quasistationary waves using 300-hPa meridional winds as in step 3 of the QRA analysis procedure.

5) As in step 4 of the QRA analysis procedure, we combine the results from steps 3 and 4 to form lag-dependent distributions of wave-6 amplitude conditional on the existence or nonexistence of an EPV waveguide. Again, the conditional distributions can be summarized in terms of their means and standard deviations to enable a comparison of the waveguide and no-waveguide distributions.

6) We repeat steps 1 to 5 using EPV data for four other isentropes: 330, 320, 315, and 310 K. The derived relationship between EPV waveguide status and wave amplitude depends substantially on the isentrope used for the EPV waveguide analysis, as discussed in the main text.

## Supplementary Materials

### This PDF file includes:

Supplementary text Figs. S1 to S8 Tables S1 to S10 References
