# Supplementary material for: Resonant Rossby wave mechanism for extreme weather performs poorly in simple model test
Source: Sci Adv. 2026 Apr 22;12(17):eadp3054. doi: 10.1126/sciadv.adp3054 (PMC13101858; doi:10.1126/sciadv.adp3054)
Supplement: Supplementary file 1 — Supplementary text Figs. S1 to S8 Tables S1 to S10 References [file sciadv.adp3054_sm.pdf]

Science Advances
AAAS

Supplementary Materials for

Resonant Rossby wave mechanism for extreme weather performs poorly in simple model test

Todd A. Mooring and Marianna Linz

Corresponding author: Todd A. Mooring, tmooring@alum.mit.edu

Sci. Adv. **12**, eadp3054 (2026)
DOI: 10.1126/sciadv.adp3054

This PDF file includes:

Supplementary Text
Figs. S1 to S8
Tables S1 to S10
References

Supplementary Text

Wave composites

We briefly examine the three-dimensional spatial structure of the waves in figs. S3 and S4. For each experiment we display composites conditional on 300 hPa QRA waveguide status. (The window means composited here are not lagged relative to their associated waveguides—in the terminology of the detailed statement of methods, we choose $D = 0$ for this analysis.) For the `wave6_heat` experiment, we do the final cross-window averages in the conventional way (temporal averaging at fixed spatial position). However, a more sophisticated approach is needed to generate composites for the `no_heat` experiment. Since this experiment lacks the wave 6 thermal forcing of `wave6_heat`, wave 6 quasistationary waves in `no_heat` will not have any physically preferred zonal phase and therefore a conventional cross-window average would almost certainly simply smear out the waves rather than reveal their typical structure. To address this issue, we use a phase shifting technique. Prior to computing the cross-window average, we make a (window-specific but field-independent) zonal shift of all fields of interest chosen so that the wave 6 components of one particular field (the 300 hPa meridional wind, area-averaged over 37.5°–57.5°N) are in perfect phase alignment across windows. To the extent that all wave 6 quasistationary waves in `no_heat` have coherent structures in three-dimensional space and across variables, this should mitigate the phase alignment problem.

We apply these compositing techniques to four fields: surface pressure and near-surface temperature (both shown in fig. S3), and meridional wind at 300 hPa and area-averaged over 37.5°–57.5°N (both shown in fig. S4). Unsurprisingly (given the forcing used for `wave6_heat` and the compositing procedure used for `no_heat`) all of the fields have wave 6 structures. In our view the most important result from figs. S3 and S4 is the apparent insensitivity of wave structure to 300 hPa QRA waveguide status and even the presence or absence of wave 6 heating. In fact, the strong structural similarity between waves in the two experiments can be interpreted as evidence that our specified heating field efficiently excites waves—which is advantageous for and consistent with (if not necessarily sufficient for) QRA. Our argument for efficient excitation is as follows: presumably the `wave6_heat` composites show the forced response to the wave 6 specified heating, while the `no_heat` composites show a free quasistationary wave of the same zonal wavenumber. But to the extent that these waves have similar meridional and vertical structures, the specified heating therefore tends to excite a forced wave similar to the free quasistationary waves—and the concept of an oscillatory system responding strongly to forcing near the system's natural oscillatory frequencies is a familiar one throughout the physical sciences. Note also that the meridional phase tilt of the 300 hPa meridional wind waves appears somewhat larger south of the amplitude maxima for the cases where 300 hPa QRA waveguides are present (fig. S4A-D). This indicates *stronger* equatorward escape of wave activity in the presence of waveguides, which is inconsistent with the theoretical expectation that waveguides trap wave activity but consistent with the observed relationship between QRA waveguide status and wave amplitude.

Furthermore, the unambiguously wave 6 character of the `wave6_heat` waves and the relative independence of wave structure from QRA waveguide status in both experiments affirms the appropriateness of using the wave 6 component of a 300 hPa meridional mean meridional wind field as a metric of overall wave amplitude. However, note that figs. S3 and S4 themselves

probably cannot be used to make reliable comparisons of wave amplitudes between the two experiments—to the extent that the phase shifting technique that must be used to make the `no_heat` composites is imperfect, remaining phase misalignments for levels and fields other than 300 hPa meridional wind will tend to artificially damp the estimated wave amplitude. We therefore (for example) cannot determine whether the apparent weakness of the `no_heat` cyclones and anticyclones (fig. S3A-B) relative to their `wave6_heat` counterparts (fig. S3C-D) is real or merely an artifact of inadequate zonal phase alignment of the `no_heat` surface pressure fields.

Robustness of stationary wavenumber and wave amplitude results

Fig. S5 shows the 300 hPa squared stationary wavenumber and underlying zonal mean zonal wind profiles as functions of latitude for three different window lengths and for both hemispheres. Fig. S6 shows wave amplitudes as a function of lag for a variety of analysis choices. Our main result (wave suppression with 300 hPa QRA waveguides and wave enhancement without them) is robust to all tested combinations of these analysis choices, including those not shown here (e.g. 30-day windows with central tendency and variability characterized using the median and quartiles).

Permutation test analyses of the QRA waveguide–wave amplitude relationship: methods

The simple technique used in Figs. 3 and S6 to argue for a negative relationship between (300 hPa zonal wind) QRA waveguide existence and 300 hPa meridional wind zonal wavenumber 6 amplitude is feasible only because in practice our simulations provide many realizations of the mean flow both with and without such waveguides. We are able to extend this fact to the EPV waveguide analysis as well (by tuning the waveguide existence threshold), but as outlined in the main paper a similar analysis using QRA waveguides based on 200 or 500 hPa zonal winds is precluded by the relatively small number of time windows in which the mean flow at these levels satisfies the (otherwise unchanged) waveguide existence criteria. (In graphical terms, the blue lines in 200 or 500 hPa counterparts of Figs. 3 and S6 would be too heavily contaminated by internal variability noise to obtain a meaningful result.)

To address this issue, we therefore developed an alternative approach based on permutation tests *(59)* to perform 200 and 500 hPa QRA waveguide analyses (and repeat the 300 hPa QRA waveguide analysis). In brief, we define a test statistic Y that captures the difference in wave amplitudes with and without a waveguide. For a given pressure level, experiment, and hemisphere this statistic takes on the value Y^{obs}. To contextualize the value of Y^{obs}, we compare it to a null distribution for Y that describes the values Y can be expected to take in the absence of any real relationship between waveguide status and wavenumber 6 wave amplitude. The null distribution for Y is generated by computing a very large number of values of Y from the actual time series of wave 6 amplitude and a very large number of synthetic time series of waveguide existence status. These synthetic time series of waveguide existence status are in turn formed by randomly reordering the (one) actual time series of waveguide existence status, thereby severing any real statistical relationship between waveguide existence and wave amplitude encoded in Y^{obs}. A value of Y^{obs} that is extreme in the context of the Y null distribution then constitutes evidence for some sort of relationship between waveguide existence status and wave amplitude.

Let us now more precisely and rigorously describe the calculations:

1. For a given pressure level, experiment, and hemisphere of interest, we divide the available (11640 days of instantaneous 6-hourly) zonal wind data into M non-overlapping N-day windows where N is 15, 30 or 60. We compute the time mean zonal mean zonal wind for each of the M windows, and determine whether each window has a QRA waveguide as defined in the "Waveguide detection algorithms" section of the materials and methods. This process yields an M-element time series of waveguide existence status as a binary variable.
2. We also compute associated M-element time series of zonal wavenumber 6 wave amplitude. We evaluate wave amplitude using the 300 hPa meridional wind field (regardless of the level at which we evaluate the zonal wind field for the waveguide calculation) and compute the wavenumber 6 wave amplitude from a window-mean area-weighted meridional mean over 37.5°–57.5°. Not only will we analyze an M-element time series defined over the same windows as the M-element waveguide status time series (i.e., lag 0), we will also analyze wave amplitude time series lagged by $\sim N/2$ and 30 days relative to the waveguide status time series. As with the many lags tested in our main analysis, the purpose of this approach is to address the possibility that the wave amplitude takes time to respond to the waveguide status of the mean flow. For N = 15, the $\sim N/2$ lag is 8 days. (For N = 60, the $\sim N/2$ and 30-day lags are of course the same.)
3. Using the waveguide status time series from step 1, we sort the two or three M-element lists of wave 6 amplitudes from step 2 by waveguide status. We then use the sorted wave amplitudes to compute mean wave amplitudes conditional on waveguide existence and nonexistence, which we denote X_{WG}^{obs} and X_{noWG}^{obs} respectively.
4. We define the test statistic Y as the difference in mean wave 6 amplitude between windows with and without QRA waveguides. The observed value of Y (for a given zonal wind pressure level, experiment, hemisphere, and N value of interest) is $Y^{obs} = X_{WG}^{obs} - X_{noWG}^{obs}$. If waveguide existence favors (disfavors) the development of large-amplitude waves, we would expect to find $Y^{obs} > 0$ ($Y^{obs} < 0$) given a sufficiently large number of windows from which to compute X_{WG}^{obs} and X_{noWG}^{obs}.
5. Of course, we have only M windows from which to compute X_{WG}^{obs}, X_{noWG}^{obs}, and Y^{obs}. We therefore must entertain the possibility that a given Y^{obs} is also significantly influenced by sampling variability that is not reflective of causal relationships between waveguide status and wave amplitude. To address this possibility, we produce 10^4 random permutations of the M-element waveguide status time series and then use them to compute an additional 10^4 Y values (per lag) following the process outlined in steps 1 to 4.
6. Because there can be no causal connection between random permutations of the waveguide status time series and the (unpermuted, real) wave amplitude time series, the ensembles of 10^4 Y values constitute null distributions that portray what values of Y^{obs} are plausible in the absence of a real relationship between waveguide status and wave amplitude. For each Y^{obs} of interest we therefore compute P^{obs}, the percentile of the associated Y null distribution to which Y^{obs} corresponds. The normalized nature of P^{obs} makes it more useful for identifying relationships between waveguide status and wave amplitude, regardless of the waves' absolute amplitude. Given the sign convention used to define Y^{obs}, P^{obs} values near 100 would constitute evidence that QRA waveguides promote the development of large-amplitude zonal wavenumber 6 waves. In contrast,

P^{obs} values near 0 would suggest suppression of wavenumber 6 amplitude by waveguides.

7. A possible weakness of the procedure laid out in steps 1 to 6 is that the permutation process as described in step 5 does not preserve any temporal autocorrelations that may be present in waveguide status time series taken directly from our GCM. Neglect of such autocorrelation may lead to an unrealistic Y null distribution and thus biased estimates of P^{obs}. To address this issue, we also use an alternative version of the permutation process in which we consolidate the M windows into 97 non-overlapping 120-day "superwindows" such that for N = 15, 30, or 60 each superwindow consists of an 8-, 4-, or 2-element time series of waveguide status, respectively. We then generate 10^4 synthetic waveguide status time series to make the Y null distributions by permuting the 120-day superwindows. This process is akin to a block bootstrap *(59)* and all other aspects of the analysis remain the same, except that we refer to the final output statistic as P_*^{obs} rather than P^{obs}.
8. There are also a number of rather arbitrary choices about how to quantify wave amplitude made in steps 2 and 3, to which our results may be sensitive. First, we could quantify wave amplitude for an individual window at 43.25° instead of as a meridional mean. Second, the X metric of wavenumber 6 amplitude need not be a cross-window mean: we could for example also define it as the mean+1 standard deviation, the median, or the 75th percentile. We therefore perform our analyses in steps 1 to 7 using not just the aforementioned 37.5°–57.5°/cross-window mean amplitude quantification approach but also the other seven approaches implied by the choices enumerated here.

Note that our choice not to refer to P^{obs} and P_*^{obs} as "P values" is deliberate, and we are not claiming to be conducting formal null hypothesis significance tests. We make these choices for two reasons: First, the performance of multiple versions of the analysis presumably provides more opportunities to get P^{obs} and P_*^{obs} values near 0 or 100 by "chance" and therefore a claim of "significance" based on an individual P^{obs} or P_*^{obs} value would likely be overstated. However, for a given hemisphere and experiment the various P^{obs} and P_*^{obs} values cannot be independent (because they all ultimately summarize the same model output) and therefore there is no clear path to addressing this issue via a multiple tests correction.

Second, we developed this entire permutation test-based analysis concept after unsuccessfully attempting to apply our previous simple technique at 200 hPa. Thus although we have not knowingly "P hacked" *(60)* the permutation test-based analysis approach to produce any particular result, it would not be accurate to claim full independence of methods development from the data we are analyzing. We therefore engage in only qualitative interpretation of our derived P^{obs} and P_*^{obs} values, with an emphasis on robustness of results across the various analysis choices and consideration of the full range of possibilities consistent with the reported values.

<u>Permutation test analyses of the QRA waveguide–wave amplitude relationship: results</u>

We present the results of our permutation test-based analysis of the QRA waveguide–zonal wavenumber 6 wave amplitude relationship in six tables. Tables S1–S3 present all results from steps 1 to 7 of the above-described analysis workflow with one table per QRA waveguide pressure level. For each window 300 hPa meridional winds are averaged over 37.5°–57.5° and

characteristic wave amplitudes are computed as cross-window means. Tables S4–S6 summarize the additional wave amplitude quantification sensitivities investigated in step 8. Rather than reporting all eight available P^{obs} or P_*^{obs} values for a given hemisphere/experiment/window length/lag combination, we report only the minima and maxima for simplicity.

Table S1 applies our new analysis approach to the 300 hPa QRA waveguides that are the focus of the main paper. The P^{obs} values are extremely small—at most 0.06, and typically zero. The P_*^{obs} values can be larger—as much as 0.50—but are still often zero. In all cases the qualitative result is clear: the P^{obs} and P_*^{obs} values are much smaller than one would expect to obtain in the absence of a relationship between waveguide status and wave amplitude. Indeed, $P \approx 0$ suggests a clear negative relationship between waveguide existence and wave amplitude, consistent with results obtained via the simpler technique that is the main focus of our paper.

Table S2 analyzes 500 hPa QRA waveguides. Only 2 of the 56 valid P^{obs} and P_*^{obs} values exceed 50, and all are less than 60. This finding is not supportive of QRA theory, and indeed some of the P^{obs} and P_*^{obs} values are quite close to zero. However this result is not necessarily robust to the different realizations of internal variability in different hemispheres. Note also that waveguides are extremely rare for 30- and 60-day averaging windows, and that they are apparently absent for 60-day windows of the `no_heat` experiment.

The putative 200 hPa QRA waveguides are first analyzed in table S3. Results are strikingly different from those obtained for 300 hPa waveguides. The very low P^{obs} and P_*^{obs} values are now gone and in some cases replaced by values near 100—as one would expect if *200 hPa* QRA waveguides do in fact promote the development of large-amplitude waves. Results for `wave6_heat` at lags 0 and +8 days with 15-day windows are particularly impressive, and the (worse) +30 day result could be argued to have little physical significance because the lag is too large relative to the window length. Results for other window lengths and the `no_heat` experiment are generally less impressive and frequently suffer from a lack of interhemispheric robustness and/or a very small number of windows with waveguides to use in the analysis.

Tables S4 and S5 investigate the sensitivity of P^{obs} and P_*^{obs} to the various choices of how to characterize zonal wavenumber 6 wave amplitudes. Our conclusion that there is a negative relationship between 300 hPa QRA waveguide existence and wave amplitude remains highly robust (table S4). Nor are any of the P^{obs} or P_*^{obs} values from our analysis of 500 hPa QRA waveguides particularly supportive of the idea that such waveguides promote the existence of large-amplitude waves (table S5). The results for 200 hPa QRA waveguides presented in table S6 are qualitatively quite similar to those presented for our default wave amplitude quantification approach in table S3.

<u>Permutation test analyses of the EPV waveguide–wave amplitude relationship</u>
For completeness we also applied the permutation test-based analysis technique to our diagnosis of EPV waveguides—results are presented in tables S7–S10. Because the EPV waveguide analysis is performed for 10 different isentropes it is not useful to print here all of the derived P^{obs} and P_*^{obs} values. Instead, tables S7–S10 are analogous to tables S4–S6 in the sense that both groups of tables report ranges of P^{obs} and P_*^{obs} values. However, the ranges reported in tables S7–S10 are computed over not just the wave amplitude quantification methods

enumerated in step 8 of the permutation test algorithm but also over two or three isentropes and two choices (5 K and 10 K) for the size of the θ finite difference underlying the calculation of $\partial\theta/\partial p$ in equation 15. (The results shown in Fig. 4 of the main paper are based on a 5 K finite difference.)

The contents of tables S7–S10 together can be summarized as follows:

1. The existence of EPV waveguides at 330 K–355 K is associated with weaker zonal wavenumber 6 waves or has no clear relationship to the amplitude of these waves.
2. In the `wave6_heat` experiment, EPV waveguide existence at 320 K–325 K (310 K–315 K) is universally associated with weaker (stronger) zonal wavenumber 6 waves.
3. In the `no_heat` experiment, EPV waveguide existence at 320 K–325 K is generally associated with weaker waves or has no clear effect on wave amplitude depending on how exactly the analysis is performed. In contrast, (and consistent with the `wave6_heat` results) EPV waveguide existence at 310 K–315 K is typically associated with stronger zonal wavenumber 6 waves.

QRA waveguides and near-surface temperature extremes: overview and results

We investigate the relationship between 300 hPa QRA waveguide existence and near-surface temperature extremes by analyzing lowest-model-level temperature data. We search for extreme events—defined variously as below the local 1st or 5th percentiles or above the local 95th or 99th percentiles—and characterize the overall abundance of such extreme events in terms of the fraction of the area of the 37.5°–57.5° latitude band in which temperatures are extreme. We then investigate the dependence of these area fractions—our extreme event metrics—on QRA waveguide status. We perform such analyses using not only instantaneous but also window-averaged temperatures, similar to *(31)*. To test the robustness of our results to internal variability, we carry out separate analyses for each hemisphere and then compare them. A full explanation of our methodology is provided in the next section.

When we examine 15-day window-mean temperatures and k_s^2 profiles, we find that extreme (i.e., unusually hot *or* cold) temperatures are marginally less common with a 300 hPa QRA waveguide than without one (fig. S8A, gray and black lines in white columns). This is consistent with the idea that large amplitude waves are associated with surface extremes and that wave amplitudes are smaller when a waveguide is present. We also see that the sensitivity or lack thereof of total extreme event frequency to 300 hPa QRA waveguide status is the sum of opposite sensitivities of hot and cold extreme frequencies: the presence of a waveguide is associated with fewer >95th percentile and more <5th percentile extremes (fig. S8A, orange and light blue lines in white columns). These findings are robust to the definition of extreme temperature: the same qualitative patterns hold for the >99th percentile and <1st percentile events and the <1st and >99th percentile events aggregated together (fig. S8A, red, dark blue, and black lines in white columns). The analysis based on instantaneous temperatures shows almost all of the same results, except there are marginally more extreme events with a QRA waveguide than without for the `no_heat` experiment (fig. S8A, gray and black lines in gray columns). This effect is only slightly larger than the difference between hemispheres, however, and disappears in `wave6_heat`.

We repeated the analysis with 30- and 60-day windows and found that the qualitative results are extremely similar (fig. S8B-C). The only differences arise when the window length is raised to 60 days: the minor success of the 300 hPa QRA waveguide in boosting total instantaneous extreme temperature frequencies in `no_heat` disappears (gray and black lines in gray columns, fig. S8C) and time mean temperatures below the 1st percentile are no longer robustly promoted by waveguides in `wave6_heat` (dark blue lines in white columns, fig. S8C). We also performed the extremes analysis using temperatures at a single latitude (43.25°) and show the results based on 15-day windows in fig. S8D. Because QRA theory does not make predictions for changes in the frequencies of hot and cold extremes separately, in the interests of brevity we describe only the aggregated <5th or >95th percentile and <1st or >99th percentile results. The finding most consistent with QRA theory is that there are more <1st or >99th percentile instantaneous temperatures in the presence of a 300 hPa QRA waveguide for `no_heat`, regardless of the length of window (15, 30, or 60 days) used for the waveguide diagnosis. No other result concerning these two-tailed extreme temperatures is clearly supportive of QRA. Instead, such temperatures are either more frequent in the absence of a waveguide or there is simply no clear relationship between waveguide status and extreme event frequency.

Finally, for all cases where we evaluated extremes within or near QRA waveguides the presence of a waveguide was associated with less frequent extreme hot events. We found only one situation in which the presence of waveguides was associated with more frequent extreme hot events: when we analyzed extreme events equatorward of the waveguide, in the 17.5°–37.5° latitude band (fig. S8E). Total frequency of extreme events was not necessarily higher in the presence of a waveguide, however, and elevated extreme event frequencies outside waveguide regions do not lend credence to QRA theory.

<u>Waveguides and near-surface temperature extremes: detailed methods</u>
This is an expanded description of the step-by-step procedure we use to derive the near-surface temperature extreme results:

1. For the northern hemisphere of a given experiment we select temperature data from the lowest model level, covering the same span of time as the zonal wind data used for the 300 hPa QRA waveguide calculations described in the main text and in the materials and methods. Recall that this time period is divisible into M windows of length N days apiece, and since the temperature data are available as instantaneous values every 6 hours we thus have $4MN$ temperatures at every horizontal location in the model.
2. For each horizontal location, we use the $4MN$ temperature values to estimate the 95th percentile of the local temperature distribution. We classify a timestep as extreme if and only if its temperature exceeds this threshold.
3. We then divide the $4MN$ timesteps of temperature data into the same M non-overlapping windows used to search for waveguides in our analysis of (meridional wind) wave amplitudes. (We do not bother with lagged—i.e., $D \neq 0$—windows for this analysis of temperature extremes, given the similarity of the $0 \leq D \leq 30$ wave amplitude results.) For clarity, let us first consider the analysis of just one window. For each latitude and timestep in this window, we compute the fraction of longitudes at which the temperature is extreme. We then average this value across all $(4N)$ timesteps of the window to form a latitude-dependent metric of the frequency of extreme events.

4. We repeat step 3 for all M windows, creating a metric of extreme event frequency that is a function of latitude and window index i.
5. By leveraging our previously-derived results on whether or not the zonal mean flow for each of the M windows contains a QRA waveguide that should trap wave 6, we form latitude-dependent distributions of extreme event frequency conditioned upon the existence (or non-existence) of such a waveguide. By averaging across the windows contributing to each distribution, we summarize the distributions in terms of their (latitude-dependent) means.
6. To further reduce the dimensionality of our results, we take area-weighted meridional means of the latitude-dependent distributional means. This meridional averaging is analogous to the meridional averaging of the wind field used for the wave amplitude analysis and ultimately leaves us with scalar estimates of the frequency of extreme events, conditional on the existence (or non-existence) of a wave 6 QRA waveguide.
7. We want to check for a difference between the extreme event frequencies with and without a 300 hPa QRA waveguide, but the result of step 6 is a pair of scalars without uncertainty information making any difference between them difficult to interpret. We therefore generate an additional pair of extreme event conditional frequencies by repeating steps 1–6 for the southern hemisphere of the same experiment. This enables us to check the robustness to internal variability in the model of any difference we might see.
8. To explore the sensitivity of our results to the magnitude and sign of the temperature extreme examined, we repeat steps 1–7 for five other definitions of temperature extreme: above 99th percentile, below 5th percentile, below 1st percentile, below 5th or above 95th percentile, and below 1st or above 99th percentile.

The above-described extreme events analysis examines whether instantaneous values of temperature are extreme. To perform the window-averaged analysis we follow a very similar procedure to the one for instantaneous temperatures, with the following changes:

1. At step 1, we reduce each time series of $4MN$ instantaneous temperature values to M non-overlapping window mean values.
2. At step 2, we do the 95th percentile calculation and subsequent identification of extreme events using the M-element series of (N-day) window mean temperatures in place of the $4MN$-element series of instantaneous temperatures.
3. At step 3, we can therefore omit the time average because there is now only one "timestep" per window.

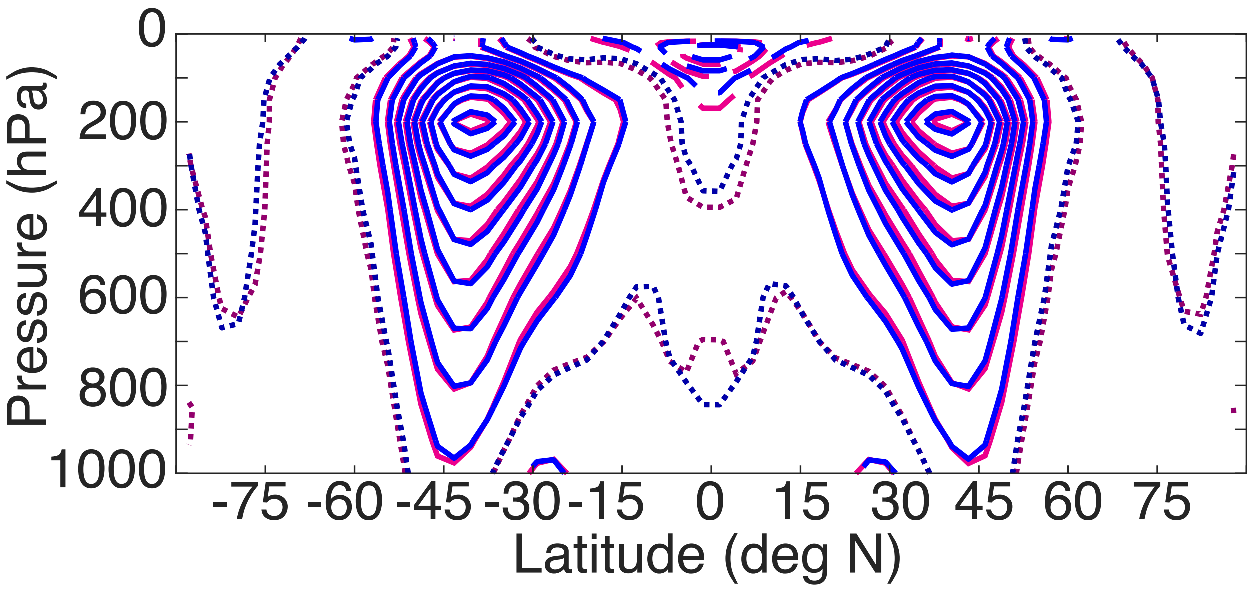

Fig. S1. Zonal mean flows for the idealized GCM experiments. Zonal mean zonal winds on pressure levels are computed over 11640 days of each model experiment. Winds for `wave6_heat` (`no_heat`) are shown in pink (blue). Contour spacing is 3 m/s, with solid (dashed) contours denoting positive (negative) velocities. The zero contour is dotted.

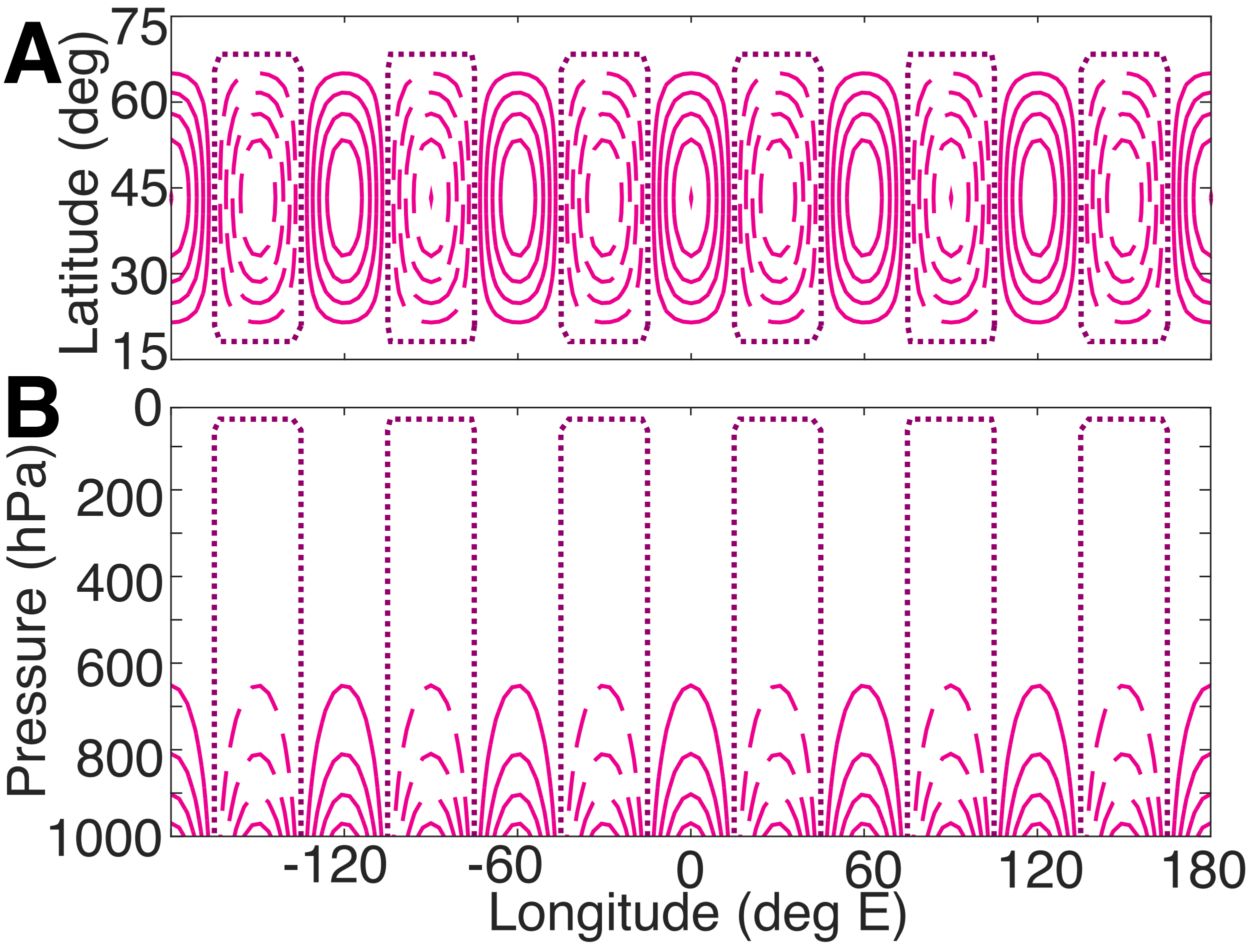

Fig. S2. Structure and strength of the specified heating field $\partial T/\partial t|_{wave6}$ used as additional forcing for `wave6_heat`. Only a single hemisphere is shown because the field is interhemispherically symmetric. The longitude-latitude section (**A**) is at 1000 hPa, while the longitude-pressure section (**B**) is an area-weighted meridional mean over 37.5°–57.5°. Contour spacing is 0.2 K/day, with positive (negative) heating rates indicated by solid (dashed) lines. The darker dotted line is the zero contour. The formula for $\partial T/\partial t|_{wave6}$ is provided as equation 20 of the main paper.

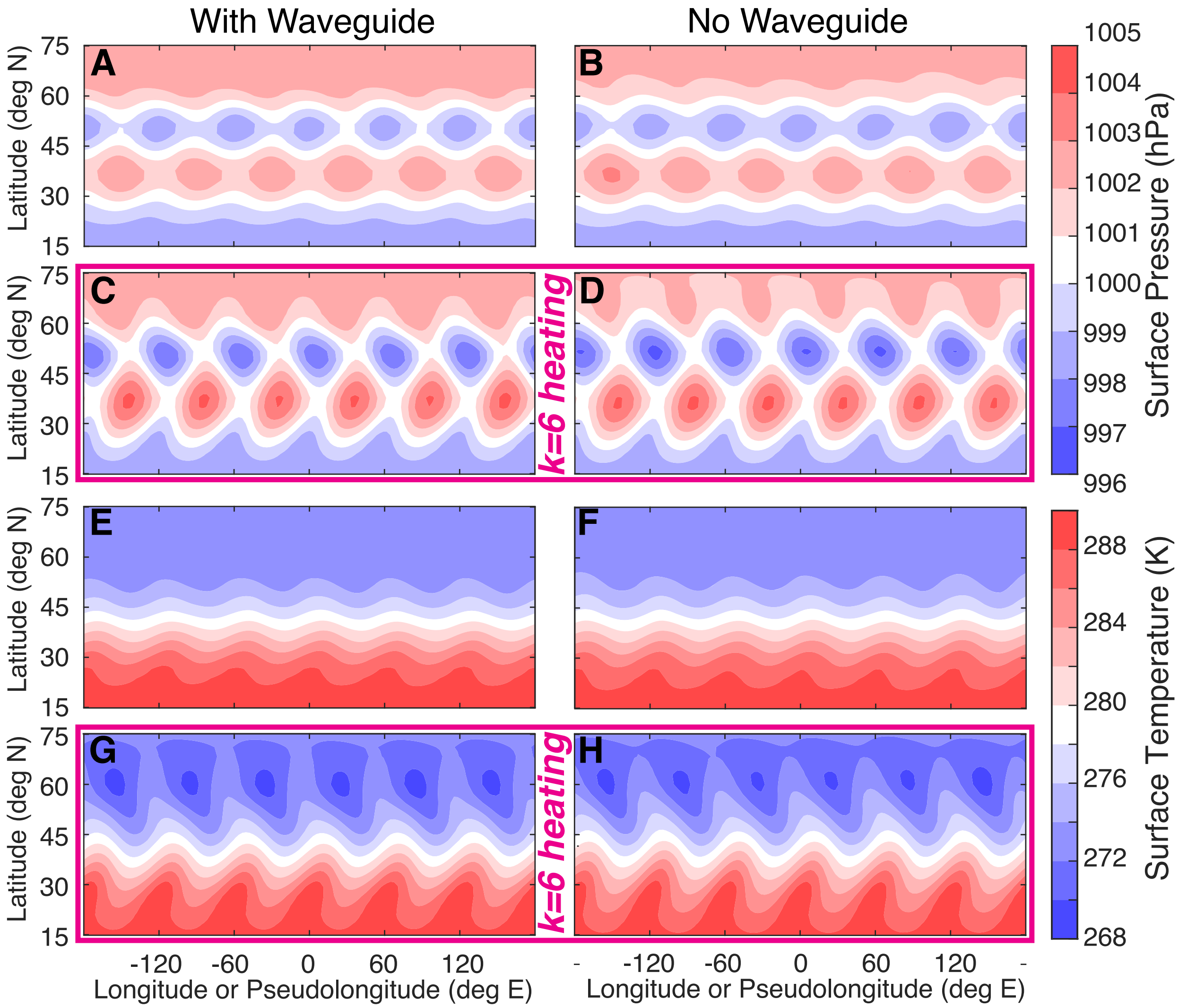

Fig. S3. Surface pressure and near-surface temperature composite maps. Average spatial patterns for surface pressure (**A–D**) and near-surface temperature (**E–H**) for windows with a 300 hPa QRA waveguide (left column) and without a 300 hPa QRA waveguide (right column) for the `no_heat` (**A**, **B**, **E**, **F**) and `wave6_heat` (**C**, **D**, **G**, **H**) experiments. Compositing for `no_heat` is described in the supplementary text, and results for this experiment are plotted as a function of phase-shifted pseudolongitude.

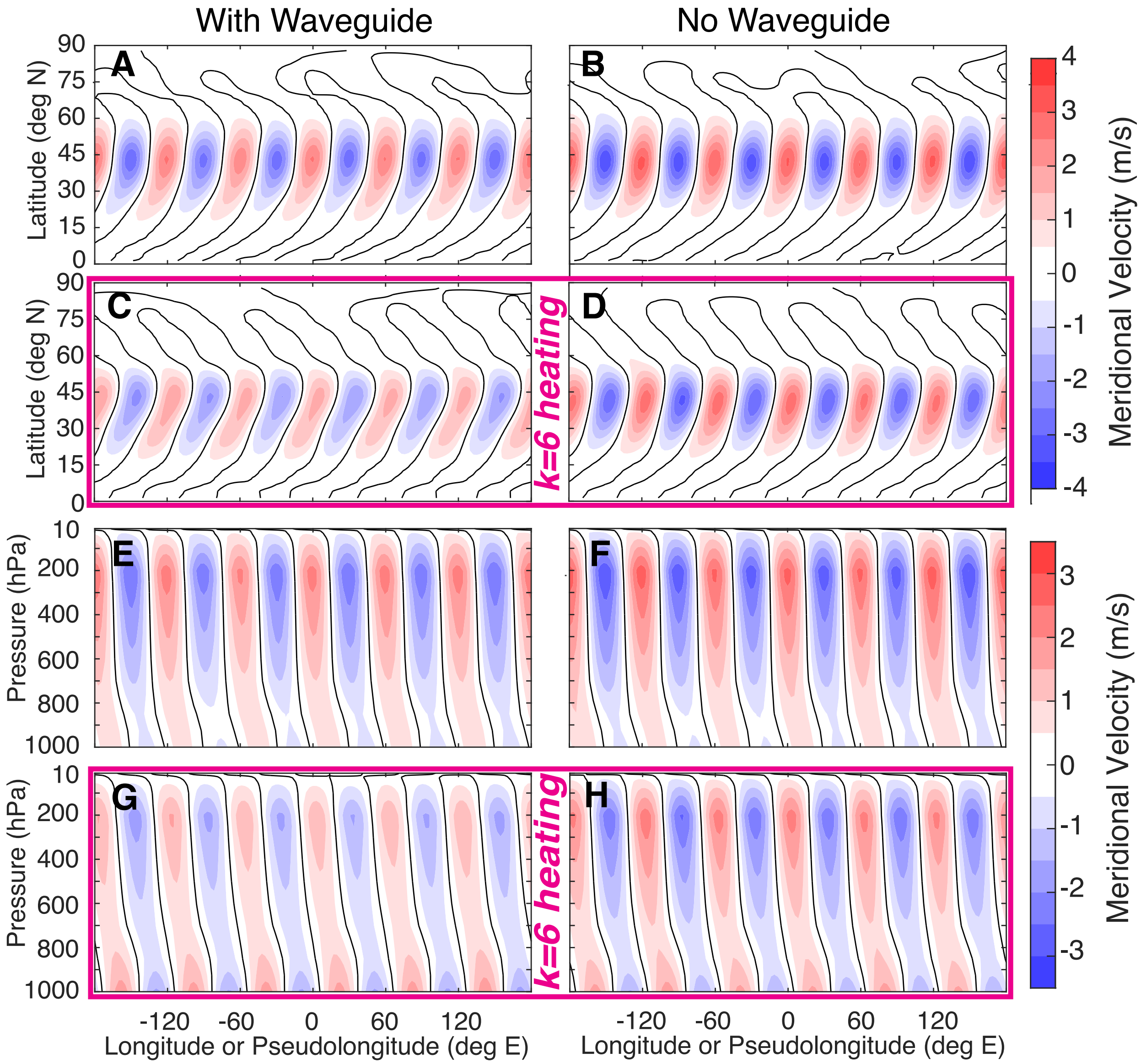

Fig. S4. Northward velocity composite maps. Average spatial patterns for the northward velocity at 300 hPa (**A–D**) and averaged between 37.5°–57.5°N (**E–H**) for windows with a 300 hPa QRA waveguide (left column) and without a 300 hPa QRA waveguide (right column) for the `no_heat` (**A**, **B**, **E**, **F**) and `wave6_heat` (**C**, **D**, **G**, **H**) experiments. Compositing for `no_heat` is described in the supplementary text, and results for this experiment are plotted as a function of phase-shifted pseudolongitude.

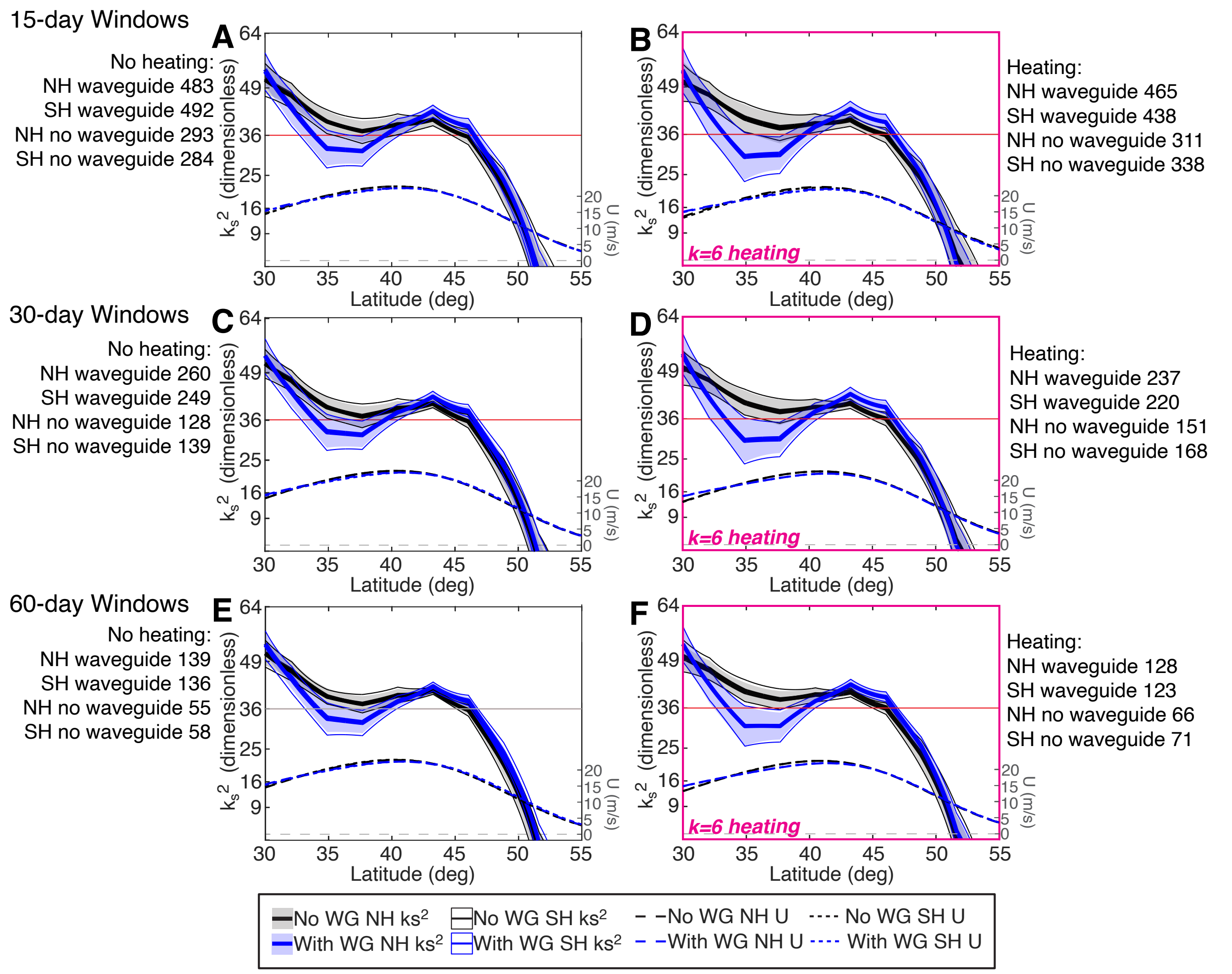

Fig. S5. Sensitivities of 300 hPa squared stationary wavenumbers and associated zonal jets to hemisphere and averaging window length. Similar to Fig. 2 of the main paper—composite k_s^2 (solid lines, left vertical axis) and zonal mean zonal wind (dashed and dotted lines, right vertical axis) profiles for windows with a 300 hPa QRA waveguide present (blue) and without a 300 hPa QRA waveguide present (black). Northern hemisphere k_s^2 composites are shown in thick solid lines, while southern hemisphere composites are shown in thinner lines which are generally indistinguishable from the thicker northern hemisphere lines. One standard deviation above and below each composite k_s^2 profile is shown with shading for the northern hemisphere and in the thinnest solid lines for the southern hemisphere. The 300 hPa zonal mean zonal wind for the northern (southern) hemisphere is shown with dashed (dotted) lines. The left (right) column shows results for the `no_heat` (`wave6_heat`) experiment. (**A**) and (**B**) show results for 15-day windows, (**C**) and (**D**) for 30-day windows, and (**E**) and (**F**) for 60-day windows. Numbers of windows for which a QRA waveguide is and is not present are listed to the side of each panel. The total number of available windows is halved with each doubling of the window length, as the analyses for the different window lengths are simply alternative partitionings of the same fixed-length model runs. Showing results for both hemispheres separately allows us to confirm that they are very similar and therefore robust to internal variability.

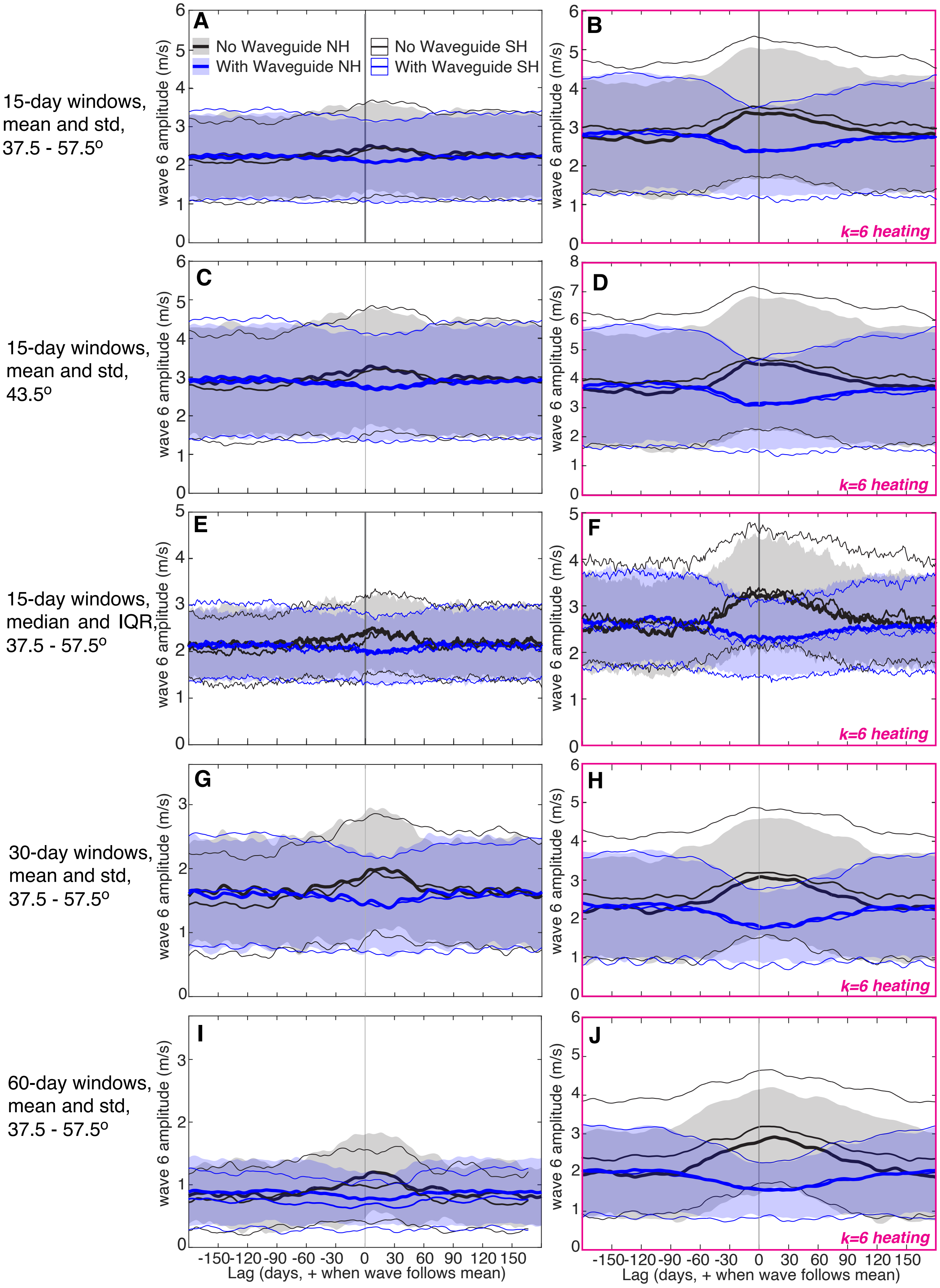

15-day windows, mean and std, 37.5 - 57.5°
15-day windows, mean and std, 43.5°
15-day windows, median and IQR, 37.5 - 57.5°
30-day windows, mean and std, 37.5 - 57.5°
60-day windows, mean and std, 37.5 - 57.5°
A
B
C
D
E
F
G
H
I
J
No Waveguide NH
With Waveguide NH
No Waveguide SH
With Waveguide SH
wave 6 amplitude (m/s)
k=6 heating
Lag (days, + when wave follows mean)

Fig. S6. Sensitivities of 300 hPa QRA waveguide–wave amplitude relationships to averaging choices. Similar to Fig. 3 of the main paper—amplitude of quasistationary zonal wavenumber 6 waves in the 300 hPa meridional wind field for the `no_heat` (left column) and `wave6_heat` (right column) experiments as a function of lag for a variety of analysis choices. Blue (black) indicates windows with (without) a 300 hPa QRA waveguide present. Northern hemisphere central tendency results are plotted with heavy lines and have uncertainty estimates shown with shading. Southern hemisphere central tendency results are plotted with lighter lines, and the lightest weight lines are used to plot southern hemisphere uncertainty estimates. (**A**) and (**B**) are the same as in Fig. 3 of the main paper except that they also include southern hemisphere results. (**C**) and (**D**) are the same as (**A**) and (**B**) but use wave amplitudes evaluated at 43.25° latitude only instead of averaged over 37.5°–57.5° latitude. (**E**) and (**F**) are the same as (**A**) and (**B**) but describe the characteristic wave amplitude and its variability using the median and interquartile range (IQR) rather than the mean and standard deviation (std). (**G**) and (**H**) are the same as (**A**) and (**B**) but use 30-day averaging windows. Finally, (**I**) and (**J**) are the same as (**A**) and (**B**) but use 60-day averaging windows.

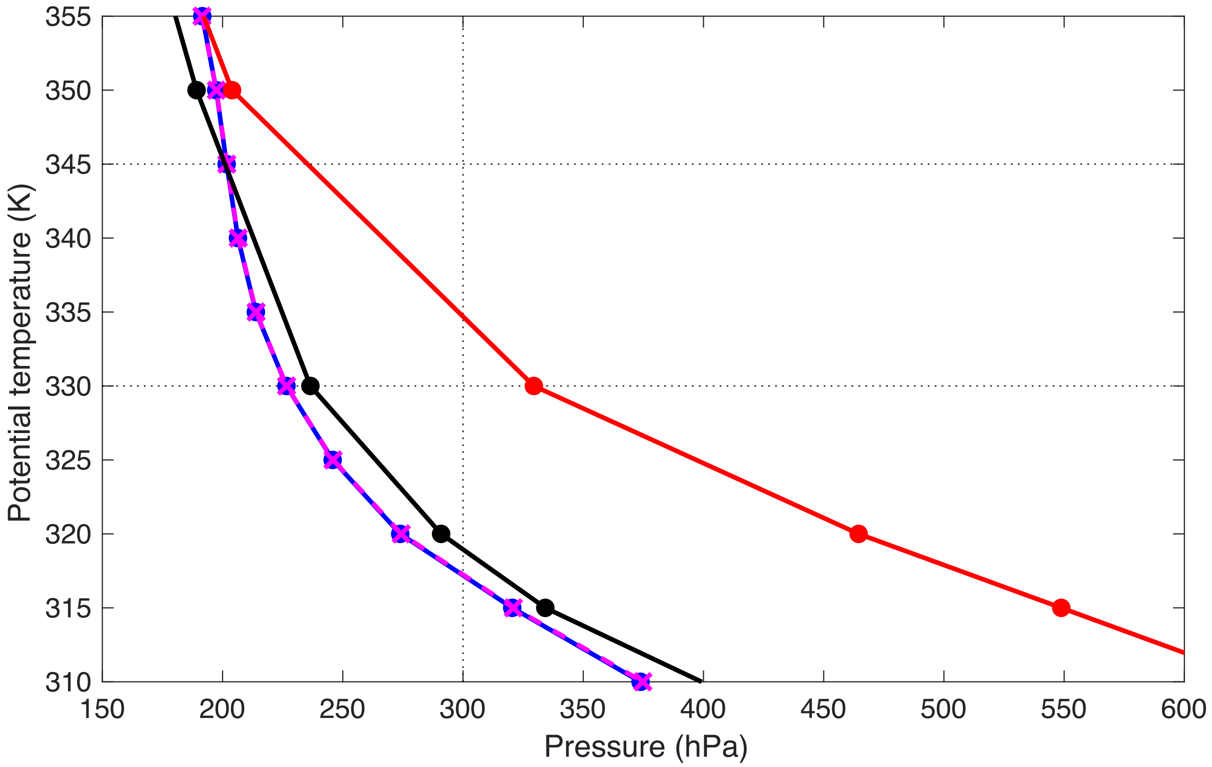

Fig. S7. Mean thermal structure in idealized GCM simulations and ERA5 northern hemisphere. Mean pressures as a function of potential temperature for 30°–70°N. Blue solid line with filled circle markers (magenta dashed line with pink X markers) shows results for the `no_heat` (`wave6_heat`) simulation, using the same 11640 days of data underpinning fig. S1. Red (black) solid line with filled circle markers shows results for ERA5 boreal summer 1995-2024 (winter 1995/6-2024/5). The idealized GCM results for 30°–70°S are negligibly different from those shown here and ERA5 decadal variability and trends are very small compared to the summer/winter difference.

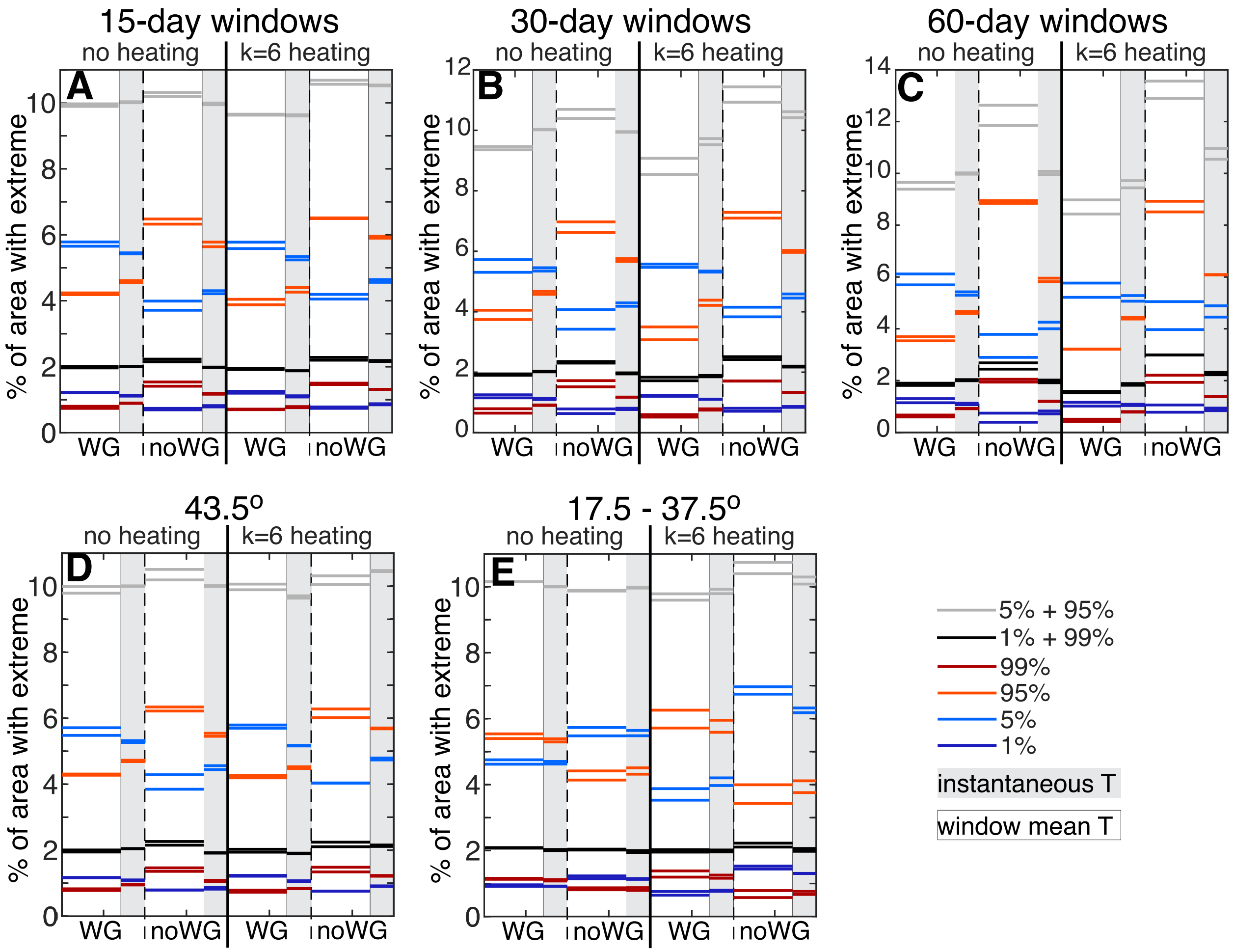

Fig. S8. Relationship between surface temperature extremes and 300 hPa QRA waveguides. Each panel is based on a different choice of time window or meridional region for quantifying extreme temperatures. Within each panel, results are presented as a function of whether wave 6 heating is used in the model, the temperature threshold(s) used to define extremes, and whether time-averaged or instantaneous temperatures are analyzed. Each panel has eight columns—the left (right) four columns apply to the `no_heat` (`wave6_heat`) experiment. For each experiment, the left/WG (right/noWG) pair of columns displays extreme event abundance in the presence (absence) of a 300 hPa QRA waveguide. For each experiment/QRA waveguide status combination, there are two columns: the white (gray) column shows results based on time-averaged (instantaneous) temperatures. Different colors of horizontal lines indicate different extreme event definitions. Abundances of cold extremes colder than the 5th (1st) percentiles of the local temperature distributions are marked in light (dark) blue. Abundances of hot extremes hotter than the 95th (99th) percentiles of the local temperature distributions are marked in orange (red). Gray (black) horizontal lines denote the combined abundances of <5th and >95th (<1st and >99th) percentile extremes. Two horizontal lines of each color—one for each hemisphere—are marked for each column. The disagreement between the two lines in a given column provides a metric of the internal variability-related uncertainty in the true extreme event abundances. (**A**) uses 15-day windows with extreme event abundance quantified using the time mean area fraction of the 37.5°–57.5° region in which temperatures are extreme. (**B**) and (**C**) are the same as (**A**) but

using 30- and 60-day windows, respectively. (**D**) quantifies temperature extremes only at 43.25° latitude, and (**E**) shows results based on 17.5°–37.5° latitude—both (**D**) and (**E**) use 15-day windows. Numbers of windows used for these calculations are listed in fig. S5.

Experiment	Window length (days)	NH WGs	SH WGs	Total windows	Lag (days)	P^{obs} NH	P^{obs} SH	P_*^{obs} NH	P_*^{obs} SH
no_heat	60	139	136	194	0	0	0	0	0
					+30	0	0	0	0
no_heat	30	260	249	388	0	0	0	0	0.02
					+15	0	0	0	0
					+30	0	0	0	0.01
no_heat	15	483	492	776	0	0	0.06	0.02	0.50
					+8	0	0	0	0
					+30	0.02	0.05	0.14	0.16
wave6_heat	60	128	123	194	0	0	0	0	0
					+30	0	0	0	0
wave6_heat	30	237	220	388	0	0	0	0	0
					+15	0	0	0	0
					+30	0	0	0	0
wave6_heat	15	465	438	776	0	0	0	0	0
					+8	0	0	0	0
					+30	0	0	0	0

Table S1. Permutation test analyses of relationship between 300 hPa QRA waveguide status and wave 6 amplitude. Wave amplitude is quantified using 300 hPa meridional winds averaged over 37.5°–57.5°, see supplementary text for further details including formal definitions and interpretations of P^{obs} and P_*^{obs}. For a given experiment and window length, the third and fourth columns give the number of windows with waveguides and the fifth column lists the total number of windows into which the simulation is divided. Red shading denotes $P \leq 5$.

**Experiment**	**Window length (days)**	**NH WGs**	**SH WGs**	**Total windows**	**Lag (days)**	P^{obs} **NH**	P^{obs} **SH**	P_*^{obs} **NH**	P_*^{obs} **SH**
no_heat	60	0	0	194	0				
					+30				
no_heat	30	1	1	388	0	13.93	12.35	14.36	13.24
					+15	7.23	4.57	9.33	4.37
					+30	34.46	3.39	35.80	3.05
no_heat	15	4	6	776	0	8.82	16.84	11.41	19.71
					+8	1.13	45.25	1.48	46.21
					+30	35.84	53.21	38.59	57.55
wave6_heat	60	1	3	194	0	30.89	0.89	34.92	1.00
					+30	15.39	0.07	13.55	0.12
wave6_heat	30	5	5	388	0	23.37	0.18	26.51	0.18
					+15	29.78	0.03	30.70	0.05
					+30	1.20	0.35	1.35	0.39
wave6_heat	15	15	16	776	0	7.56	2.22	8.95	4.46
					+8	6.79	15.00	10.63	19.64
					+30	0.65	1.07	1.34	1.96

Table S2. Permutation test analyses of relationship between 500 hPa QRA waveguide status and wave 6 amplitude. As Table S1, but with mean state zonal winds evaluated at 500 hPa. Pink shading denotes $5 < P \leq 10$. P^{obs} and P_*^{obs} cannot be computed for the no_heat experiment with 60-day windows because no waveguides exist.

Experiment	Window length (days)	NH WGs	SH WGs	Total windows	Lag (days)	P^{obs} NH	P^{obs} SH	P_*^{obs} NH	P_*^{obs} SH
no_heat	60	0	1	194	0		96.52		94.73
					+30		98.08		96.74
no_heat	30	5	4	388	0	73.21	70.56	79.11	68.33
					+15	24.64	92.78	30.86	89.10
					+30	23.78	91.98	27.14	88.48
no_heat	15	12	12	776	0	98.98	63.56	99.06	63.84
					+8	99.61	59.75	99.47	60.98
					+30	92.91	76.51	89.91	75.28
wave6_heat	60	2	2	194	0	90.65	75.90	90.76	74.22
					+30	70.57	63.42	69.39	61.32
wave6_heat	30	7	10	388	0	88.48	61.71	88.83	60.01
					+15	92.95	81.75	90.11	78.43
					+30	37.29	86.97	34.34	84.60
wave6_heat	15	37	34	776	0	83.39	95.23	82.90	92.17
					+8	98.54	98.68	97.75	96.58
					+30	43.22	91.13	46.93	86.31

Table S3. Permutation test analyses of relationship between 200 hPa QRA waveguide status and wave 6 amplitude. As Table S1, but with mean state zonal winds evaluated at 200 hPa. Blue (light blue) shading denotes $P \geq 95$ ($90 \leq P < 95$).

Experiment	Window length (days)	Total windows	Lag (days)	Hem	Number WGs	p^{obs} Min	p^{obs} Max	p^{obs}_{*} Min	p^{obs}_{*} Max
no_heat	60	194	0	N	139	0	0.01	0	0.03
			0	S	136	0	0.03	0	0.03
			+30	N	139	0	0.24	0	0.28
			+30	S	136	0	0.02	0	0.01
no_heat	30	388	0	N	260	0	0	0	0
			0	S	249	0	0.02	0	0.03
			+15	N	260	0	0	0	0
			+15	S	249	0	0	0	0
			+30	N	260	0	0	0	0
			+30	S	249	0	0.01	0	0.08
no_heat	15	776	0	N	483	0	0.03	0	0.16
			0	S	492	0	1.49	0.12	3.75
			+8	N	483	0	0	0	0.01
			+8	S	492	0	0	0	0.09
			+30	N	483	0	0.15	0.05	0.66
			+30	S	492	0.01	0.30	0.02	0.77
wave6_heat	60	194	0	N	128	0	0	0	0
			0	S	123	0	0	0	0
			+30	N	128	0	0	0	0
			+30	S	123	0	0	0	0
wave6_heat	30	388	0	N	237	0	0	0	0
			0	S	220	0	0	0	0
			+15	N	237	0	0	0	0
			+15	S	220	0	0	0	0
			+30	N	237	0	0	0	0
			+30	S	220	0	0	0	0

wave6_heat	15	776	0	N	465	0	0	0	0
			0	S	438	0	0	0	0
			+8	N	465	0	0	0	0
			+8	S	438	0	0	0	0
			+30	N	465	0	0	0	0
			+30	S	438	0	0	0	0

Table S4. Sensitivity of 300 hPa QRA waveguide permutation test results to wave amplitude evaluation method. Similar to table S1, but investigates sensitivity of results to the method of quantifying the amplitude of the zonal wavenumber 6 waves. See supplementary text for details, including an explanation of the eight quantification methods over which the P^{obs} and P_*^{obs} minima and maxima are computed.

Experiment	Window length (days)	Total windows	Lag (days)	Hem	Number WGs	P^{obs} Min	P^{obs} Max	P_*^{obs} Min	P_*^{obs} Max
no_heat	60	194	0	N	0				
			0	S	0				
			+30	N	0				
			+30	S	0				
no_heat	30	388	0	N	1	13.79	13.93	13.26	14.36
			0	S	1	12.35	15.23	13.24	14.42
			+15	N	1	6.94	7.23	9.33	11.69
			+15	S	1	4.57	5.53	4.37	5.41
			+30	N	1	31.61	34.46	31.59	35.80
			+30	S	1	3.39	3.76	1.93	3.05
no_heat	15	776	0	N	4	4.54	27.83	6.45	32.79
			0	S	6	2.50	22.51	3.00	22.99
			+8	N	4	0.24	3.80	0.51	4.97
			+8	S	6	10.30	58.76	11.10	57.89
			+30	N	4	19.20	37.22	21.11	41.05
			+30	S	6	31.90	71.44	37.21	73.48
wave6_heat	60	194	0	N	1	30.67	30.89	34.92	36.94
			0	S	3	0.66	4.52	0.69	4.89
			+30	N	1	15.39	18.48	13.55	16.33
			+30	S	3	0.01	0.52	0.02	0.53
wave6_heat	30	388	0	N	5	14.61	39.11	16.46	41.17
			0	S	5	0.03	3.99	0.10	3.48
			+15	N	5	18.11	43.14	18.27	42.45
			+15	S	5	0.01	0.61	0.02	0.60
			+30	N	5	1.00	5.98	1.28	5.78
			+30	S	5	0.16	2.35	0.19	2.66

wave6_heat	15	776	0	N	15	1.71	23.40	1.50	25.72
			0	S	16	0.76	15.94	2.26	19.36
			+8	N	15	6.49	47.74	10.20	51.14
			+8	S	16	1.33	15.00	2.49	19.64
			+30	N	15	0.04	4.07	0.20	6.88
			+30	S	16	0.46	17.27	1.14	18.43

Table S5. Sensitivity of 500 hPa QRA waveguide permutation test results to wave amplitude evaluation method. As table S4, but with mean state zonal winds evaluated at 500 hPa.

Experiment	Window length (days)	Total windows	Lag (days)	Hem	Number WGs	P^{obs} Min	P^{obs} Max	P_*^{obs} Min	P_*^{obs} Max
no_heat	60	194	0	N	0				
			0	S	1	95.58	96.52	92.49	94.73
			+30	N	0				
			+30	S	1	98.08	98.08	96.74	96.74
no_heat	30	388	0	N	5	39.88	79.06	49.37	81.31
			0	S	4	58.48	89.32	56.83	86.56
			+15	N	5	15.40	35.13	20.48	39.99
			+15	S	4	85.49	92.78	82.17	89.10
			+30	N	5	10.66	53.87	12.40	54.77
			+30	S	4	90.21	94.96	86.43	92.29
no_heat	15	776	0	N	12	87.06	99.16	90.94	99.23
			0	S	12	33.62	63.56	39.88	65.02
			+8	N	12	95.13	99.93	96.76	99.81
			+8	S	12	29.46	80.20	36.90	80.24
			+30	N	12	80.49	95.28	75.12	93.79
			+30	S	12	68.56	89.36	68.26	87.36
wave6_heat	60	194	0	N	2	87.42	99.99	87.45	99.95
			0	S	2	75.55	82.07	73.66	81.63
			+30	N	2	70.41	94.05	69.30	98.44
			+30	S	2	54.11	63.89	53.15	61.75
wave6_heat	30	388	0	N	7	87.37	96.39	86.97	95.92
			0	S	10	51.59	73.28	50.71	72.49
			+15	N	7	92.30	96.08	90.11	94.19
			+15	S	10	57.81	92.33	53.67	89.88
			+30	N	7	33.05	52.88	31.16	47.39
			+30	S	10	62.34	86.97	61.80	84.60

wave6_heat	15	776	0	N	37	53.31	91.56	55.55	90.49
			0	S	34	85.44	99.36	83.79	98.79
			+8	N	37	96.04	98.54	95.01	97.75
			+8	S	34	91.52	99.74	85.95	99.12
			+30	N	37	3.55	91.44	3.48	90.46
			+30	S	34	55.28	99.63	51.29	98.66

Table S6. Sensitivity of 200 hPa QRA waveguide permutation test results to wave amplitude evaluation method. As table S4, but with mean state zonal winds evaluated at 200 hPa.

Experiment	Window length (days)	Total windows	Lag (days)	Hem	Number WGs	P^{obs} Min	P^{obs} Max	P^{obs}_{*} Min	P^{obs}_{*} Max
no_heat	60	194	0	N	139	0.59	62.78	0.67	63.37
			0	S	136	3.14	89.31	2.84	92.80
			+30	N	139	0	66.06	0	65.40
			+30	S	136	0.04	49.40	0.03	49.39
no_heat	30	388	0	N	260	0	34.77	0	35.39
			0	S	249	0.03	85.35	0.03	85.73
			+15	N	260	0	86.25	0	87.94
			+15	S	249	0	40.06	0	44.36
			+30	N	260	0	78.67	0	79.31
			+30	S	249	0	74.63	0	76.10
no_heat	15	776	0	N	483	0	0.21	0	0.36
			0	S	492	0	3.38	0	3.22
			+8	N	483	0	21.67	0	24.21
			+8	S	492	0	0.90	0	1.94
			+30	N	483	0	68.86	0	69.23
			+30	S	492	0	23.80	0	28.89
wave6_heat	60	194	0	N	128	0	22.60	0	22.11
			0	S	123	0	2.68	0	2.57
			+30	N	128	0	1.36	0	1.58
			+30	S	123	0	0.99	0	1.19
wave6_heat	30	388	0	N	237	0	0	0	0
			0	S	220	0	0.01	0	0.10
			+15	N	237	0	2.71	0	2.59
			+15	S	220	0	0	0	0.01
			+30	N	237	0	1.64	0	1.91

			+30	S	220	0	0.52	0	0.68
`wave6_heat`	15	776	0	N	465	0	0.01	0	0.03
			0	S	438	0	0	0	0
			+8	N	465	0	2.95	0	4.27
			+8	S	438	0	0.01	0	0
			+30	N	465	0	4.52	0	4.84
			+30	S	438	0	0.03	0	0.13

Table S7. Permutation test results for EPV waveguides defined at 345, 350, and 355 K. Similar to table S4, but with waveguides defined using Ertel PV on isentropes and the associated P^{obs} and P_*^{obs} minima and maxima taken over all three isentropes, two $\partial\theta/\partial p$ layer thicknesses, and eight wave amplitude quantification methods.

Experiment	Window length (days)	Total windows	Lag (days)	Hem	Number WGs	P^{obs} Min	P^{obs} Max	P_*^{obs} Min	P_*^{obs} Max
no_heat	60	194	0	N	139	1.19	83.33	1.31	83.88
			0	S	136	34.23	99.30	35.06	99.31
			+30	N	139	0.49	48.42	0.40	48.16
			+30	S	136	53.79	99.42	52.10	99.27
no_heat	30	388	0	N	260	0.22	69.72	0.34	69.26
			0	S	249	24.19	92.72	25.74	90.87
			+15	N	260	0.10	84.96	0.10	81.15
			+15	S	249	13.18	98.30	14.76	97.39
			+30	N	260	0.99	94.35	0.92	92.92
			+30	S	249	6.89	96.53	7.02	95.39
no_heat	15	776	0	N	483	0	64.89	0	57.99
			0	S	492	1.06	65.90	2.33	66.92
			+8	N	483	0.07	56.74	0.16	57.06
			+8	S	492	0.26	52.21	0.54	52.44
			+30	N	483	1.69	99.85	2.41	99.57
			+30	S	492	5.34	80.87	7.06	77.83
wave6_heat	60	194	0	N	128	0	47.67	0	47.88
			0	S	123	0	73.89	0	73.70
			+30	N	128	0	11.52	0	11.61
			+30	S	123	0	28.31	0	27.78
wave6_heat	30	388	0	N	237	0	16.14	0	20.16
			0	S	220	0	2.02	0	2.32
			+15	N	237	0	70.59	0	61.84
			+15	S	220	0	0.06	0	0.08
			+30	N	237	0	22.64	0	20.71

			+30	S	220	0	28.53	0	30.48
wave6_heat	15	776	0	N	465	0	0.04	0	0.20
			0	S	438	0	0	0	0
			+8	N	465	0	15.53	0	19.37
			+8	S	438	0	0	0	0.04
			+30	N	465	0	8.24	0.01	7.93
			+30	S	438	0	0.64	0	1.52

Table S8. Permutation test results for EPV waveguides defined at 330, 335, and 340 K. As table S7, but instead based on the 330, 335, and 340 K isentropes.

Experiment	Window length (days)	Total windows	Lag (days)	Hem	Number WGs	P^{obs} Min	P^{obs} Max	P_*^{obs} Min	P_*^{obs} Max
no_heat	60	194	0	N	139	0	11.87	0	11.96
			0	S	136	0	49.23	0	53.06
			+30	N	139	0	1.07	0	1.03
			+30	S	136	0	90.02	0	89.99
no_heat	30	388	0	N	260	0	0.35	0	0.54
			0	S	249	0	21.03	0	21.73
			+15	N	260	0	1.07	0	1.14
			+15	S	249	0	3.31	0	4.09
			+30	N	260	0	3.13	0	2.85
			+30	S	249	0	5.61	0	6.16
no_heat	15	776	0	N	483	0	19.90	0	18.54
			0	S	492	0	6.18	0	5.62
			+8	N	483	0	4.10	0	6.09
			+8	S	492	0	3.94	0	4.00
			+30	N	483	0	27.95	0	24.82
			+30	S	492	0	3.75	0	6.25
wave6_heat	60	194	0	N	128	0	0.64	0	0.58
			0	S	123	0	0	0	0
			+30	N	128	0	0.02	0	0.01
			+30	S	123	0	0	0	0
wave6_heat	30	388	0	N	237	0	0	0	0
			0	S	220	0	0	0	0
			+15	N	237	0	2.27	0	1.36
			+15	S	220	0	0	0	0
			+30	N	237	0	0.59	0	0.48

			+30	S	220	0	0	0	0
wave6_heat	15	776	0	N	465	0	0	0	0
			0	S	438	0	0	0	0
			+8	N	465	0	0	0	0
			+8	S	438	0	0	0	0
			+30	N	465	0	0.01	0	0.01
			+30	S	438	0	0	0	0

Table S9. Permutation test results for EPV waveguides defined at 320 and 325 K. As table S7, but instead based on the 320 and 325 K isentropes.

**Experiment**	**Window length (days)**	**Total windows**	**Lag (days)**	**Hem**	**Number WGs**	P^{obs} **Min**	P^{obs} **Max**	P_*^{obs} **Min**	P_*^{obs} **Max**
no_heat	60	194	0	N	139	91.09	99.99	90.97	100.00
			0	S	136	99.59	100.00	99.47	100.00
			+30	N	139	63.33	99.99	64.11	99.98
			+30	S	136	84.98	100.00	85.77	100.00
no_heat	30	388	0	N	260	100.00	100.00	100.00	100.00
			0	S	249	99.94	100.00	99.88	100.00
			+15	N	260	100.00	100.00	99.99	100.00
			+15	S	249	100.00	100.00	99.99	100.00
			+30	N	260	100.00	100.00	99.99	100.00
			+30	S	249	99.98	100.00	99.98	100.00
no_heat	15	776	0	N	483	99.76	100.00	99.19	100.00
			0	S	492	99.87	100.00	99.57	100.00
			+8	N	483	99.90	100.00	99.73	100.00
			+8	S	492	96.19	100.00	94.41	100.00
			+30	N	483	96.69	100.00	94.02	100.00
			+30	S	492	99.36	100.00	98.32	100.00
wave6_heat	60	194	0	N	128	100.00	100.00	100.00	100.00
			0	S	123	100.00	100.00	100.00	100.00
			+30	N	128	100.00	100.00	100.00	100.00
			+30	S	123	100.00	100.00	100.00	100.00
wave6_heat	30	388	0	N	237	100.00	100.00	100.00	100.00
			0	S	220	100.00	100.00	100.00	100.00
			+15	N	237	100.00	100.00	100.00	100.00
			+15	S	220	100.00	100.00	100.00	100.00
			+30	N	237	100.00	100.00	100.00	100.00

			+30	S	220	100.00	100.00	100.00	100.00
wave6_heat	15	776	0	N	465	100.00	100.00	100.00	100.00
			0	S	438	100.00	100.00	100.00	100.00
			+8	N	465	100.00	100.00	99.99	100.00
			+8	S	438	100.00	100.00	100.00	100.00
			+30	N	465	100.00	100.00	100.00	100.00
			+30	S	438	100.00	100.00	100.00	100.00

Table S10. Permutation test results for EPV waveguides defined at 310 and 315 K. As table S7, but instead based on the 310 and 315 K isentropes.

REFERENCES

1. S. Campbell, T. A. Remenyi, C. J. White, F. H. Johnston, Heatwave and health impact research: A global review. *Health Place* **53**, 210–218 (2018).

2. K. L. Ebi, A. Capon, P. Berry, C. Broderick, R. de Dear, G. Havenith, Y. Honda, R. S. Kovats, W. Ma, A. Malik, N. B. Morris, L. Nybo, S. I. Seneviratne, J. Vanos, O. Jay, Hot weather and heat extremes: Health risks. *Lancet* **398**, 698–708 (2021).

3. D. D. Breshears, J. B. Fontaine, K. X. Ruthrof, J. P. Field, X. Feng, J. R. Burger, D. J. Law, J. Kala, G. E. St, J. Hardy, Underappreciated plant vulnerabilities to heat waves. *New Phytol.* **231**, 32–39 (2021).

4. H. Xu, J. Xiao, Z. Zhang, Heatwave effects on gross primary production of northern mid-latitude ecosystems. *Environ. Res. Lett.* **15**, 074027 (2020).

5. S. Heinicke, K. Frieler, J. Jägermeyr, M. Mengel, Global gridded crop models underestimate yield responses to droughts and heatwaves. *Environ. Res. Lett.* **17**, 044026 (2022).

6. M. R. Alizadeh, J. T. Abatzoglou, J. F. Adamowski, J. P. Prestemon, B. Chittoori, A. Akbari Asanjan, M. Sadegh, Increasing heat-stress inequality in a warming climate. *Earths Future* **10**, e2021EF002488 (2022).

7. T. Chakraborty, A. Hsu, D. Manya, G. Sheriff, Disproportionately higher exposure to urban heat in lower-income neighborhoods: A multi-city perspective. *Environ. Res. Lett.* **14**, 105003 (2019).

8. S. Perkins-Kirkpatrick, S. Lewis, Increasing trends in regional heatwaves. *Nat. Commun.* **11**, 3357 (2020).

9. D. I. V. Domeisen, E. A. B. Eltahir, E. M. Fischer, R. Knutti, S. E. Perkins-Kirkpatrick, C. Schär, S. I. Seneviratne, A. Weisheimer, H. Wernli, Prediction and projection of heatwaves. *Nat. Rev. Earth Environ.* **4**, 36–50 (2023).

10. G. J. Van Oldenborgh, M. F. Wehner, R. Vautard, F. E. L. Otto, S. I. Seneviratne, P. A. Stott, G. C. Hegerl, S. Y. Philip, S. F. Kew, Attributing and projecting heatwaves is hard: We can do better. *Earths Future* **10**, e2021EF002271 (2022).

11. R. Vautard, J. Cattiaux, T. Happé, J. Singh, R. Bonnet, C. Cassou, D. Coumou, F. D'Andrea, D. Faranda, E. Fischer, A. Ribes, S. Sippel, P. Yiou, Heat extremes in Western Europe increasing faster than simulated due to atmospheric circulation trends. *Nat. Commun.* **14**, 6803 (2023).

12. K. A. McKinnon, A. Rhines, M. P. Tingley, P. Huybers, The changing shape of Northern Hemisphere summer temperature distributions. *J. Geophys. Res. Atmos.* **121**, 8849–8868 (2016).

13. D. Argüeso, A. Di Luca, S. E. Perkins-Kirkpatrick, J. P. Evans, Seasonal mean temperature changes control future heat waves. *Geophys. Res. Lett.* **43**, 7653–7660 (2016).

14. E. A. Barnes, Revisiting the evidence linking Arctic amplification to extreme weather in midlatitudes. *Geophys. Res. Lett.* **40**, 4734–4739 (2013).

15. M. Linz, G. Chen, Z. Hu, Large-scale atmospheric control on non-gaussian tails of midlatitude temperature distributions. *Geophys. Res. Lett.* **45**, 9141–9149 (2018).

16. L.-A. Kautz, O. Martius, S. Pfahl, J. G. Pinto, A. M. Ramos, P. M. Sousa, T. Woollings, Atmospheric blocking and weather extremes over the Euro-Atlantic sector—A review. *Weather Clim. Dyn.* **3**, 305–336 (2022).

17. L. Brunner, N. Schaller, J. Anstey, J. Sillmann, A. K. Steiner, Dependence of present and future European temperature extremes on the location of atmospheric blocking. *Geophys. Res. Lett.* **45**, 6311–6320 (2018).

18. A. B. Pezza, P. van Rensch, W. Cai, Severe heat waves in Southern Australia: Synoptic climatology and large scale connections. *Clim. Dyn.* **38**, 209–224 (2012).

19. M. Röthlisberger, O. Martius, Quantifying the local effect of Northern Hemisphere atmospheric blocks on the persistence of summer hot and dry spells. *Geophys. Res. Lett.* **46**, 10101–10111 (2019).

20. E. Neal, C. S. Y. Huang, N. Nakamura, The 2021 Pacific Northwest heat wave and associated blocking: Meteorology and the role of an upstream cyclone as a diabatic source of wave activity. *Geophys. Res. Lett.* **49**, e2021GL097699 (2022).

21. N. Nakamura, C. S. Y. Huang, Atmospheric blocking as a traffic jam in the jet stream. *Science* **361**, 42–47 (2018).

22. T. Woollings, D. Barriopedro, J. Methven, S.-W. Son, O. Martius, B. Harvey, J. Sillmann, A. R. Lupo, S. Seneviratne, Blocking and its response to climate change. *Curr. Clim. Change Rep.* **4**, 287–300 (2018).

23. R. Schiemann, P. Athanasiadis, D. Barriopedro, F. Doblas-Reyes, K. Lohmann, M. J. Roberts, D. V. Sein, C. D. Roberts, L. Terray, P. L. Vidale, Northern Hemisphere blocking simulation in current climate models: evaluating progress from the Climate Model Intercomparison Project Phase 5 to 6 and sensitivity to resolution. *Weather Clim. Dyn.* **1**, 277–292 (2020).

24. B. Hoskins, T. Woollings, Persistent extratropical regimes and climate extremes. *Curr. Clim. Change Rep.* **1**, 115–124 (2015).

25. P. W. Chan, J. L. Catto, M. Collins, Heatwave–blocking relation change likely dominates over decrease in blocking frequency under global warming. *Npj Clim. Atmos. Sci.* **5**, (2022).

26. L. Stadtherr, D. Coumou, V. Petoukhov, S. Petri, S. Rahmstorf, Record Balkan floods of 2014 linked to planetary wave resonance. *Sci. Adv.* **2**, e1501428 (2016).

27. T. V. Lakshmi Kumar, G. P. Durga, K. K. Rao, H. Barbosa, A. Kulkarni, S. Patwardhan, R. K. Mall, V. B. Rao, Connection of quasi-resonant amplification to the delay in atmospheric residence times over India. *Front. Earth Sci.* **9**, 10.3389/feart.2021.615325 (2021).

28. V. Petoukhov, S. Petri, K. Kornhuber, K. Thonicke, D. Coumou, H. J. Schellnhuber, Alberta wildfire 2016: Apt contribution from anomalous planetary wave dynamics. *Sci. Rep.* **8**, 12375 (2018).

29. V. Petoukhov, S. Rahmstorf, S. Petri, H. J. Schellnhuber, Quasiresonant amplification of planetary waves and recent Northern Hemisphere weather extremes. *Proc. Natl. Acad. Sci. U.S.A.* **110**, 5336–5341 (2013).

30. I. M. Held, M. Ting, H. Wang, Northern winter stationary waves: Theory and modeling. *J. Clim.* **15**, 2125–2144 (2002).

31. D. Coumou, V. Petoukhov, S. Rahmstorf, S. Petri, H. J. Schellnhuber, Quasi-resonant circulation regimes and hemispheric synchronization of extreme weather in boreal summer. *Proc. Natl. Acad. Sci. U.S.A.* **111**, 12331–12336 (2014).

32. D. Coumou, K. Kornhuber, J. Lehmann, V. Petoukhov, "Weakened flow, persistent circulation, and prolonged weather extremes in boreal summer," in *Climate Extremes: Patterns and Mechanisms* (American Geophysical Union, 2017).

33. K. Kornhuber, V. Petoukhov, S. Petri, S. Rahmstorf, D. Coumou, Evidence for wave resonance as a key mechanism for generating high-amplitude quasi-stationary waves in boreal summer. *Clim. Dyn.* **49**, 1961–1979 (2017).

34. K. Kornhuber, S. Osprey, D. Coumou, S. Petri, V. Petoukhov, S. Rahmstorf, L. Gray, Extreme weather events in early summer 2018 connected by a recurrent hemispheric wave-7 pattern. *Environ. Res. Lett.* **14**, 054002 (2019).

35. X. Li, M. E. Mann, M. F. Wehner, S. Rahmstorf, S. Petri, S. Christiansen, J. Carrillo, Role of atmospheric resonance and land–atmosphere feedbacks as a precursor to the June 2021 Pacific Northwest Heat Dome event. *Proc. Natl. Acad. Sci. U.S.A.* **121**, e2315330121 (2024).

36. K. Kornhuber, D. Coumou, E. Vogel, C. Lesk, J. F. Donges, J. Lehmann, R. M. Horton, Amplified Rossby waves enhance risk of concurrent heatwaves in major breadbasket regions. *Nat. Clim. Change* **10**, 48–53 (2020).

37. M. E. Mann, S. Rahmstorf, K. Kornhuber, B. A. Steinman, S. K. Miller, S. Petri, D. Coumou, Projected changes in persistent extreme summer weather events: The role of quasi-resonant amplification. *Sci. Adv.* **4**, eaat3272 (2018).

38. Y. He, X. Zhu, Z. Sheng, M. He, Resonant waves play an important role in the increasing heat waves in northern hemisphere mid-latitudes under global warming. *Geophys. Res. Lett.* **50**, e2023GL104839 (2023).

39. R. H. White, K. Kornhuber, O. Martius, V. Wirth, From atmospheric waves to heatwaves: A waveguide perspective for understanding and predicting concurrent, persistent, and extreme extratropical weather. *Bull. Am. Meteorol. Soc.* **103**, E923–E935 (2022).

40. V. Wirth, Waveguidability of idealized midlatitude jets and the limitations of ray tracing theory. *Weather Clim. Dyn.* **1**, 111–125 (2020).

41. O. Martius, C. Schwierz, H. C. Davies, Tropopause-level waveguides. *J. Atmos. Sci.* **67**, 866–879 (2010).

42. C. Polster, V. Wirth, A new atmospheric background state to diagnose local waveguidability. *Geophys. Res. Lett.* **50**, e2023GL106166 (2023).

43. R. H. White, L. M. Admasu, Temporally and zonally varying atmospheric waveguides—Climatologies and connections to quasi-stationary waves. *Weather Clim. Dyn.* **6**, 549–570 (2025).

44. I. M. Held, "Stationary and quasi-stationary eddies in the extratropical troposphere: Theory," in *Large-Scale Dynamical Processes in the Atmosphere* (Academic Press, 1983), pp. 127–168.

45. H. Hersbach, B. Bell, P. Berrisford, S. Hirahara, A. Horányi, J. Muñoz-Sabater, J. Nicolas, C. Peubey, R. Radu, D. Schepers, A. Simmons, C. Soci, S. Abdalla, X. Abellan, G. Balsamo, P. Bechtold, G. Biavati, J. Bidlot, M. Bonavita, G. De Chiara, P. Dahlgren, D. Dee, M. Diamantakis, R. Dragani, J. Flemming, R. Forbes, M. Fuentes, A. Geer, L. Haimberger, S. Healy, R. J. Hogan, E. Hólm, M. Janisková, S. Keeley, P. Laloyaux, P. Lopez, C. Lupu, G.

Radnoti, P. De Rosnay, I. Rozum, F. Vamborg, S. Villaume, J. Thépaut, The ERA5 global reanalysis. *Q. J. Roy. Meteorol. Soc.* **146**, 1999–2049 (2020).

46. C. I. Garfinkel, N. Harnik, The non-Gaussianity and spatial asymmetry of temperature extremes relative to the storm track: the role of horizontal advection. *J. Clim.* **30**, 445–464 (2016).

47. P. A. O'Gorman, The effective static stability experienced by eddies in a moist atmosphere. *J. Atmos. Sci.* **68**, 75–90 (2011).

48. E. K. M. Chang, An idealized nonlinear model of the Northern Hemisphere winter storm tracks. *J. Atmos. Sci.* **63**, 1818–1839 (2006).

49. T. Ambrizzi, B. J. Hoskins, Stationary Rossby-wave propagation in a baroclinic atmosphere. *Q. J. Roy. Meteorol. Soc.* **123**, 919–928 (1997).

50. B. J. Hoskins, T. Ambrizzi, Rossby wave propagation on a realistic longitudinally varying flow. *J. Atmos. Sci.* **50**, 1661–1671 (1993).

51. G. Branstator, Circumglobal teleconnections, the jet stream waveguide, and the North Atlantic oscillation. *J. Clim.* **15**, 1893–1910 (2002).

52. V. Wirth, C. Polster, The problem of diagnosing jet waveguidability in the presence of large-amplitude eddies. *J. Atmos. Sci.* **78**, 3137–3151 (2021).

53. S. O. Guimarães, M. E. Mann, S. Rahmstorf, S. Petri, B. A. Steinman, D. J. Brouillette, S. Christiansen, X. Li, Increased projected changes in quasi-resonant amplification and persistent summer weather extremes in the latest multimodel climate projections. *Sci. Rep.* **14**, 21991 (2024).

54. C. Huntingford, D. Mitchell, K. Kornhuber, D. Coumou, S. Osprey, M. Allen, Assessing changes in risk of amplified planetary waves in a warming world. *Atmos. Sci. Lett.* **20**, e929 (2019).

55. X. Li, M. E. Mann, M. F. Wehner, S. Christiansen, Increased frequency of planetary wave resonance events over the past half-century. *Proc. Natl. Acad. Sci. U.S.A.* **122**, e2504482122 (2025).

56. I. M. Held, M. J. Suarez, A proposal for the intercomparison of the dynamical cores of atmospheric general circulation models. *Bull. Am. Meteorol. Soc.* **75**, 1825–1830 (1994).

57. P. J. Valdes, B. J. Hoskins, Linear stationary wave simulations of the time-mean climatological flow. *J. Atmos. Sci.* **46**, 2509–2527 (1989).

58. V. Petoukhov, S. Petri, S. Rahmstorf, D. Coumou, K. Kornhuber, H. J. Schellnhuber, Role of quasiresonant planetary wave dynamics in recent boreal spring-to-autumn extreme events. *Proc. Natl. Acad. Sci. U.S.A.* **113**, 6862–6867 (2016).

59. D. S. Wilks, "Frequentist statistical inference," in *Statistical Methods in the Atmospheric Sciences* (Academic Press, 2011) DOI: 10.1016/B978-0-12-385022-5.00005-1.

60. B. Tsang, *P* hacking—Five ways it could happen to you. *Nature*, 10.1038/d41586-025-01246-1 (2025).
